# Genotype imputation from low-coverage data for medical and population genetic analyses

**DOI:** 10.1101/gr.280175.124

**Published:** 2025-09

**Authors:** Simone Andrea Biagini, Sara Becelaere, Mio Aerden, Tatjana Jatsenko, Laurens Hannes, Philip Van Damme, Jeroen Breckpot, Koenraad Devriendt, Bernard Thienpont, Joris Robert Vermeesch, Isabelle Cleynen, Toomas Kivisild

**Affiliations:** 1 Department of Human Genetics, KU Leuven, Leuven 3000, Belgium;; 2 Department of Archaeology and Museology, Masaryk University, 662 43 Brno, Czech Republic;; 3 Center of Molecular Medicine, Central European Institute of Technology, Masaryk University, 662 43 Brno, Czech Republic;; 4 Center for Human Genetics, University Hospitals Leuven, University of Leuven, Leuven 3000, Belgium;; 5 Laboratory of Neurobiology, Neuroscience Department, KU Leuven, Leuven 3000, Belgium;; 6 Neurology Department, University Hospitals Leuven, Leuven 3000, Belgium;; 7 Estonian Biocentre, Institute of Genomics, University of Tartu, Tartu 51010, Estonia

## Abstract

Genotype imputation from low-pass sequencing data presents unique opportunities for genomic analyses but comes with specific challenges. In this study, we explore the impact of quality filters on genetic ancestry and Polygenic Score (PGS) estimation after imputing 32,769 low-pass genome-wide sequences (LPS) from noninvasive prenatal screening (NIPS) with an average autosomal sequence depth of ∼0.15×. In studies involving ultra-low coverage sequences, conventional approaches to secure genotype accuracy may fail, especially when multiple samples are pooled. To enhance the proportion of high-quality genotypes in large data sets, we introduce a filtering approach called GDI that combines genotype probability (GP), alternate allele dosage (DS), and INFO score filters. We demonstrate that the imputation tools QUILT and GLIMPSE2 achieve similar accuracy, which is high enough for broad-scale ancestry mapping but insufficient for high resolution principal component analysis (PCA), when applied without filters. With the GDI approach, we can achieve quality that is adequate for such purposes. Furthermore, we explored the impact of imputation errors, choice of variants, and filtering methods on PGS prediction for height in 1911 subjects with height data. We show that polygenic scores predict 23.7% of variance in height in our imputed data and that, contrary to the effect on PCA, the GDI filter does not improve the performance of PGS in height prediction. These results highlight that imputed LPS data can be leveraged for further biomedical and population genetic use, but there is a need to consider each downstream analysis tool individually for its imputation quality thresholds and filtering requirements.

Until recently, genotyping arrays were the only cost-effective approach for generating data for medical genomics at the scale of tens of thousands to millions of individuals. With decreasing costs of genome sequencing, low-pass (at coverage <1×) whole-genome sequencing followed by imputation has become a cost-effective alternative for trait mapping and estimation of polygenic scores (PGSs) (Martin et al. 2021; Wasik et al. 2021). For evolutionary and population genetic studies, sequencing of large numbers of individuals at low depth provides a more comprehensive representation of variation in the population than sequencing of a small number of individuals at a higher depth (Fumagalli 2013). Importantly, imputation performed at sufficient accuracy can offer opportunities to reuse large volumes of already generated data. Here, we explore the prospects to leverage hundreds of thousands of LPS genomes, generated from cell-free DNA (cfDNA) during noninvasive prenatal screening (NIPS) (Wang et al. 2021; Van Riel et al. 2023), for the study of maternal genotypes via imputation.

Noninvasive prenatal screening has become an integral part of prenatal care in many countries, although adoption rates vary significantly across Europe. In most European countries, fewer than 25% of individuals adopt NIPS, and in many cases, the rate is below 5% (Gadsbøll et al. 2020). However, countries such as Italy, Spain, Austria, the Netherlands, and Belgium demonstrate higher uptake. Belgium, in particular, stands out due to its progressive reimbursement policy for NIPS, which has facilitated its widespread accessibility and seamless integration into prenatal care (Bayindir et al. 2015; Vermeesch et al. 2016). Approximately 80% of NIPS performed in Belgium rely on low-pass genome-wide sequencing, making this technology a cornerstone of prenatal care in the country. More than 200,000 LPS genomes have been generated so far for NIPS in Belgium alone.

Imputation of maternal genotypes from NIPS data can offer unique genomic medicine opportunities beyond fetal aneuploidy screening at the population level, such as genome-wide association studies (GWAS) and PGS profiling of large cohorts. In this study, we focus on imputation strategies scalable to large cohorts that ensure sufficient accuracy for downstream purposes by exploring suitable postimputation filtering strategies. In our study, we demonstrate how LPS data from NIPS can be repurposed to support diverse biological analyses, showcasing the broader potential of cfDNA-derived LPS data.

Several tools enable efficient and accurate genotype imputation from LPS data. Among these, Beagle (Browning et al. 2018) and Gencove's loimpute software (Wasik et al. 2021) have been some of the most popular examples. However, they present limitations in scalability for large cohorts and increased computational costs with large reference panels. Recently, imputation methods like GLIMPSE (Rubinacci et al. 2021), GLIMPSE2 (Rubinacci et al. 2023), and QUILT (Davies et al. 2021), scalable for imputation tasks of tens of thousands of low-coverage genomes, have been developed and benchmarked against other tools, showing high accuracy and low computation costs with reference panels of tens of thousands individuals or more.

The minimum coverage from which accurate genotype imputation can be achieved may depend on various factors, including the sample quality (read length, contamination, damage), variant frequency and representation in the reference panel, and composition and size of reference panels, as well as the error sensitivity of downstream analytical methods used. Gamba et al. (2014) showed that imputation of common variants from European ancient genomes of 1× coverage can be performed at 99% accuracy when filtering out a small proportion of variants that have low genotype probability (GP < 0.99). Accuracy higher than 90% in downsampled ancient genomes was achievable also from 0.1× coverage with the cost of filtering out more variants (Hui et al. 2020). Using the 1000 Genomes Project (1kGP) reference panel and GLIMPSE, Sousa Da Mota et al. (2023) showed that the GP ≥ 0.99 filter retained only 20%–43% of the correctly imputed variants per individual sample in low coverage samples. Individual-level filtering will thus produce, even in small sample sizes, substantial cumulative missingness leading to a drastic reduction in the number of sites available for downstream analyses. In search of the optimal balance between data loss and quality with large batches of data, we explore different filtering options and quality metrics of individuals and variants at the batch level. To do so, we implemented a filtering strategy called GDI that incorporates filters on different postimputation metrics: the posterior genotype probability, the alternate allele dosage, and the INFO score, a value to measure the imputation accuracy of each imputed variant that some tools for imputation usually report (Marchini and Howie 2010).

Genotypes imputed without reference panels from NIPS sequence data from large cohorts of Chinese individuals have been used for the study of population structure in China, for replicating and finding new genome-wide associations with height, BMI, twinning, and gestational diabetes, and for calculation of polygenic scores (Liu et al. 2018, 2024; Homburger et al. 2019; Zhen et al. 2024). Similarly, a GWAS approach applied on imputed LPS data of 140,000 NIPS profiles from the Netherlands has been successful in identifying loci that affect the concentration and fragmentation properties of cell-free DNA in plasma (Linthorst et al. 2024). However, the full potential of this approach is yet to be revealed. Because of the availability of large haplotype reference panels, such as the Haplotype Reference Consortium (HRC) (The Haplotype Reference Consortium 2016) with 27,165 individuals, imputation of European samples can be achieved with increased accuracy (Davies et al. 2021; Rubinacci et al. 2021, 2023). Yet, the impact of imputation errors, particularly in the low minor allele frequency (MAF) classes, on downstream population genetic applications, such as PCA, or medical applications, such as PGS analysis, is not fully known. A PGS estimates an individual's genetic risk to develop a certain complex trait or disease by combining trait-associated variants into one predictive score. Adult height is a complex trait that is highly polygenic in nature, with 12,111 independent single nucleotide polymorphisms (SNPs) collectively accounting for 40%–45% of phenotypic variance in populations of European ancestry (Yengo et al. 2022). Its high heritability, together with the fact that height is easily measured, makes it an attractive model trait to evaluate the usability of LPS data with different imputation options for genome-wide association scanning and PGS calculation. Here, we assess the accuracy of PGS calculation from low-coverage imputed NIPS data for height and investigate the effect of different filtering strategies, including the GDI filter on the correlation between predicted and measured trait values.

In this study, we test the performance of QUILT (Davies et al. 2021) in genotype imputation on large batches (>1000 individuals) of LPS data (also referred to as NIPS data in the text) with an average coverage of ∼0.15×. As small insertions and deletions can be imputed with lower accuracy (Nguyen et al. 2024), require separate handling (Rubinacci et al. 2023), and are omitted by QUILT (Davies et al. 2021), we focus our analyses only on single nucleotide substitutions. We impute the genotypes of 32,769 Belgian individuals and test the effect of different reference panels and filtering steps on imputation accuracy of a single individual or a batch of many individuals. Using the GDI approach, we evaluate the effects of the imputation quality filters on downstream analyses, including principal component analysis (PCA) and PGS.

## Results

### Imputation accuracy from LPS data with QUILT and GLIMPSE2

We first evaluated the performance of QUILT (Davies et al. 2021) on LPS data through sensitivity values across multiple MAF bins for sites imputed as homozygous for the reference (REF) or the alternate allele (ALT) and for heterozygous sites (HET) that tend to be more challenging to impute (Ausmees et al. 2022). Additionally, we also assessed data filtered for maximum genotype probability (max(GP) ≥ 0.99), a commonly employed approach to enhance the quality of imputed data (Gamba et al. 2014; Hui et al. 2020), especially effective when working with few or individual samples. We observed (Supplemental Fig. S1) that the average sensitivity of raw imputed data across three test samples varies among different MAF bins, with clear improvements after applying a GP filter.

As detailed in Supplemental Table S1, the HET sensitivity shows an average improvement of 9.3% across all observed MAF bins after GP filtering that allows sensitivity values to surpass ∼97% for common variants (MAF > 0.05). Similarly, the ALT sensitivity, which already exhibits values exceeding ∼93% across all MAF bins in raw imputed results, demonstrates an average improvement of 3.33%, reaching values higher than ∼97% across all MAF bins post-GP filter application. Moreover, the REF sensitivity presents an average improvement of 1.8%, with values surpassing ∼98% across all MAF bins following GP filter application.

To provide a comparison with other available imputation tools, we also imputed the three test samples with GLIMPSE (Rubinacci et al. 2021) and GLIMPSE2 (Rubinacci et al. 2023). The comparison between QUILT and GLIMPSE (Supplemental Fig. S2A) highlighted that QUILT provides a higher number of correctly imputed genotypes (especially for heterozygous genotypes) after the application of a GP filter max(GP) ≥ 0.99 (Supplemental Table S2). We observe that sensitivity values show superior performance in QUILT for MAF bins below 5%, whereas for MAF bins exceeding 5%, QUILT maintains an average sensitivity value of 0.9895 (SD ± 0.28) across the three test samples. This is lower than with GLIMPSE, which presents an average sensitivity value of 0.994 (SD ± 0.14) in the same frequency category (MAF > 5%). Therefore, whereas GLIMPSE is, on average, providing an additional 0.0045 sensitivity for the MAF bin including common variants and a lower number of correctly imputed genotypes in any of the observed MAF bins, QUILT presents a better compromise between the number of correctly imputed genotypes and their quality. In the comparison with GLIMPSE2 (Supplemental Fig. S2B), we observe that, in each genotype category, both tools tend to display similar trends, both for the raw imputed and the GP-filtered data. Furthermore, we also tested a dual-step imputation with Beagle 5 (Browning et al. 2018) being the second imputation step after applying GLIMPSE or GLIMPSE2. With this test, we wanted to see whether using Beagle 5 after running GLIMPSE or GLIMPSE2 could increase the quality of the already imputed data. Overall, we observed that a dual-step imputation (Supplemental Fig. S2C,D), with a GP filter after the first step, resulted in an increased number of correctly imputed genotypes but at the expense of reduced sensitivity compared to QUILT.

**Figure 1.**
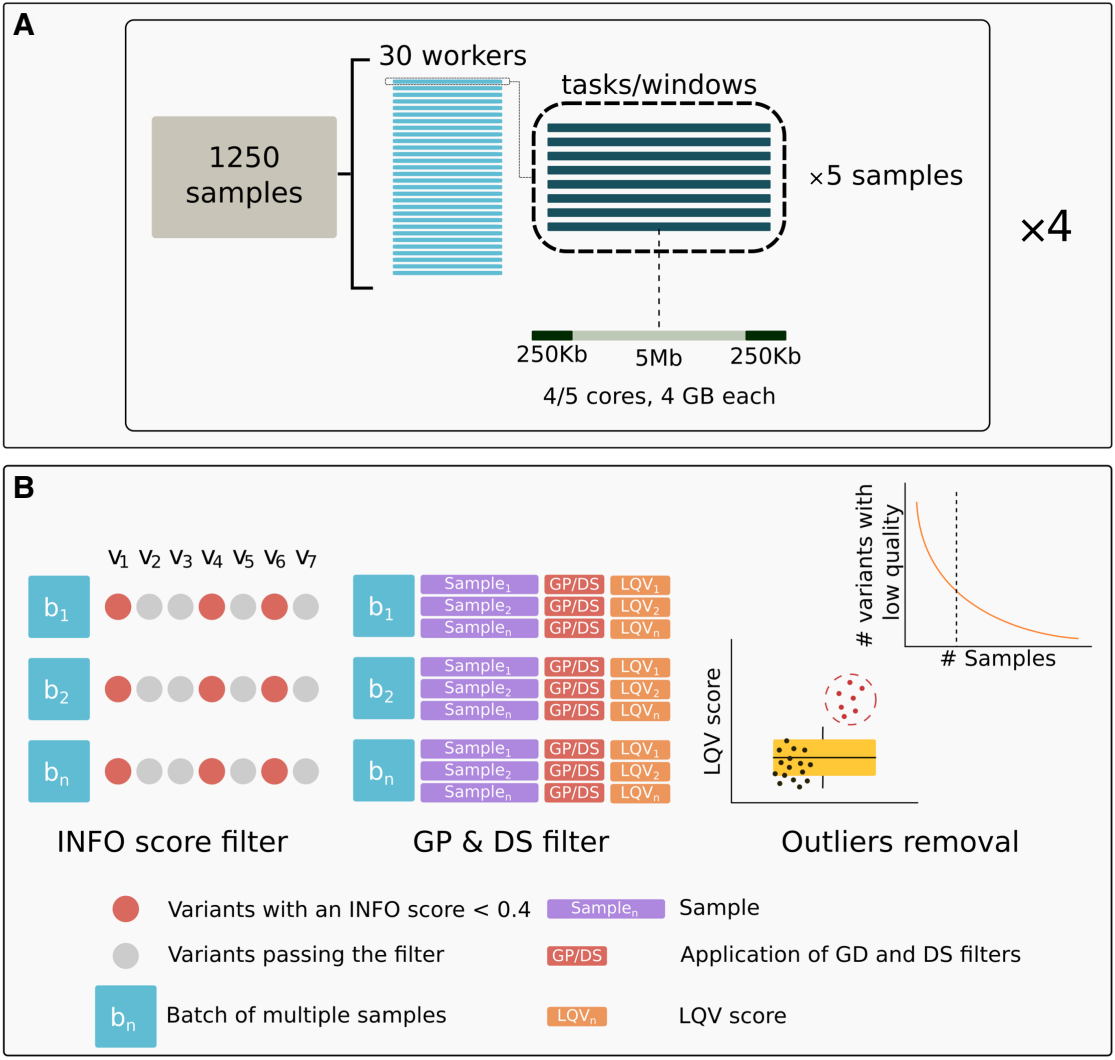
Imputation pipeline and filtering scheme used in this study. (*A*) Imputation pipeline with QUILT. The image illustrates the design of imputation of a batch of 1250 individuals, described in further detail in the Methods section. (*B*) GDI filtering scheme. INFO score filter: Each column (e.g., v1, v2, v3, etc.) represents a distinct variant, and each row (e.g., b1, b2, bn) corresponds to a batch. Variants with an INFO score <0.4 in all batches (red dots) are excluded from subsequent steps. This exclusion applies only when the same variant consistently displays this condition across all batches (e.g., three out of seven variants in the example cartoon are excluded). Variants that pass the filter (gray dots) proceed to the next step. GP & DS filter: Using the variants that passed the previous step, for each individual within a batch, an LQV score (low-quality variant score) is calculated, indicating the proportion of variants that do not meet both GP and DS thresholds. Each sample's LQV score is then used to assess the quality of the data. Outliers removal: The distribution of LQV scores is analyzed to identify and remove outlier samples (represented by the red dots in the box plot). For the remaining samples (represented by the black dots in the boxplot), the distribution of variants with low quality is assessed relative to the total number of samples. The curve in the *top right* subpanel illustrates how a cutoff is determined to exclude variants that consistently exhibit low quality in more than a specified percentage of samples. Visual inspection of UMAP plots was used to determine the optimal cutoff (additional details are provided in the main text).

Based on these results, we found that, among all the options tested, QUILT and GLIMPSE2 presented the highest performance levels, showing similar quality outcomes. Additionally, we evaluated the effectiveness of a GP filter in enhancing sensitivity values for various MAF bins and genotype categories at an individual level. However, GLIMPSE2 was not yet available at the time of data production, and QUILT was chosen for the analysis of the bulk of the data. For details on the methodology, please refer to the Methods section. The pipeline design we employed is presented in Figure 1A.

In terms of computational efficiency, a comparative analysis of QUILT and GLIMPSE2 showed that the imputation of one NIPS genome on a single core with QUILT, using a reference panel that had been preprocessed, took 21 h and 38 min, compared to 11 h and 23 min with GLIMPSE2 (a ratio of approximately 1:2). This difference is smaller than the results reported by Rubinacci et al. (2023), who observed an ∼sixfold difference in computation cost in favor of GLIMPSE2 when using the HRC panel.

An additional observation drawn from our tests is that, regardless of the imputation tool used, a negative correlation is consistently observed between fetal fraction (SeqFF) and sensitivity (Supplemental Fig. S3A), whereas a positive correlation exists between coverage and sensitivity (Supplemental Fig. S3B).

### Selection of the reference panel

We compared the effect of different reference panels on imputation accuracy of NIPS data with QUILT, using the three test samples. Specifically, we compared the 1000 Genomes Project reference panel of 2504 individuals and 32,140,179 variants and the HRC panel of 27,165 individuals and 36,258,911 variants (Table 1; Supplemental Table S3). Whereas the sensitivities of heterozygote calls of common variants (MAF > 0.05) are similarly high (>0.965) with both panels, we found that the use of the bigger HRC panel enabled us to retain more correctly imputed common variants. In total, >65,000 heterozygous variants passed the GP ≥ 0.99 filter with HRC among variants with MAF > 0.05, which is ∼5% more than with the 1000 Genomes Project panel. In line with this, the total number of missing genotype calls was greater after the GP ≥ 0.99 filters with the 1000 Genomes Project panel.

**Table 1.** Average imputation sensitivities of heterozygous genotypes obtained using HRC and 1kGP reference panels

		HET sensitivity		Number of HETs		Missing HETs	
Filter	MAF bin	HRC	1kGP	HRC	1kGP	HRC	1kGP
No filter	0.001–0.01	52.7	50.9	17,188	14,039	0	0
	0.01–0.05	78.8	74.0	83,397	77,676	0	0
	0.05–0.1	88.0	84.1	127,130	120,522	0	0
	0.1–0.3	91.9	88.5	677,999	647,528	0	0
	>0.3	93.7	90.4	789,923	755,478	0	0
max(GP) ≥ 0.99	0.001–0.01	54.2	50.4	5046	5669	71.5	59.3
	0.01–0.05	93.7	88.1	48,631	46,943	50.9	49.3
	0.05–0.1	97.7	96.1	88,139	82,656	37.6	40
	0.1–0.3	98.8	98.3	479,079	452,659	34.3	37
	>0.3	99.3	99.1	559,099	531,081	33.2	35.9
GDI	0.001–0.01	na	na	na	na	0	na
	0.01–0.05	na	na	na	na	0	na
	0.05–0.1	93.1	na	101,500	na	0	na
	0.1–0.3	96.0	na	432,852	na	0	na
	>0.3	97.4	na	417,039	na	0	na

HET sensitivity: heterozygote genotype sensitivities are expressed in percentage.

Number of HETs: number of correctly imputed heterozygous genotypes in the given MAF bin#.

Missing HETs: proportion of imputed heterozygous genotypes that were set to missing by the GP ≥ 0.99 filter in the given MAF bin.

### Cumulative missingness

Although the GP filter applied at the individual level can clearly help to enhance imputation accuracy, it introduces cumulative missingness when combining individual data. With large batches, the cumulative missingness can result in retention of only a small number, if any, of the sites that have GP value ≥0.99. Supplemental Figure S4A shows the distribution of variants removed by the GP filter per sample, highlighting the high degree of variance in the batch of imputed NIPS samples, with the majority of the individuals having 20%–50% of the variants removed, with an average of ∼30%. In the full set of 32,769 NIPS individuals, we found there are no variants that pass the GP filter in all samples. The minimum proportion of missing calls per variant we observed was 0.007 (210 individuals with GP < 0.99), with the distribution of the missingness by variants per bins of individuals not passing the filter shown in Supplemental Figure S4B.

To address the issue of cumulative missingness, we attempted first to filter out variants with an INFO score below 0.4 across all imputed batches, but this proved insufficient in removing the batch effect from high-level PCA, where we observed the clustering together of all our imputed NIPS batches separately from the reference data (Supplemental Fig. S5A). Increasing the threshold to an INFO score below 0.6 did not improve the result (Supplemental Fig. S5B). Hence, we adopted a more refined approach we call GDI filtering, that removes only the most problematic sites by integrating multiple postimputation quality metrics, including posterior genotype probability, alternate allele dosage, and the INFO score from QUILT (Fig. 1B). We applied GDI filtering dynamically on our data to optimize the maximum data quality with minimal loss of variants, observing a range of metrics of imputation accuracy in light of the performance of downstream analyses tools, PCA, and PGS.

### Data filtering

#### Exclusion of related and duplicate samples

We used QUILT to impute 32,769 samples in batches of a maximum of 1250 individuals (Fig. 1A), as detailed in the Methods section. The first step after imputation was to identify and remove duplicates and related individuals with the aim of avoiding possible allele frequency biases in the downstream analyses. Duplicates may result from separate tests for different or the same pregnancies. For the identification and removal of duplicates and closer than third degree-related individuals, we utilized IBIS (Seidman et al. 2020), which employs the same bounds as KING (Manichaikul et al. 2010), for the degree of relatedness. A kinship coefficient value of 0.088 defines the level of relatedness expected when members of the same family are within the same healthcare system (Johnson et al. 2022). However, we applied a stricter threshold, considering any pair of individuals with a kinship coefficient above 0.044 (up to third-degree relatives) as related. Notably, when cross-checking our results with additional metadata obtained at a later stage of the study, we found that our results with IBIS matched more than 90% of the recorded information related to duplicates and relatives.

#### GDI validation

**Figure 2.**
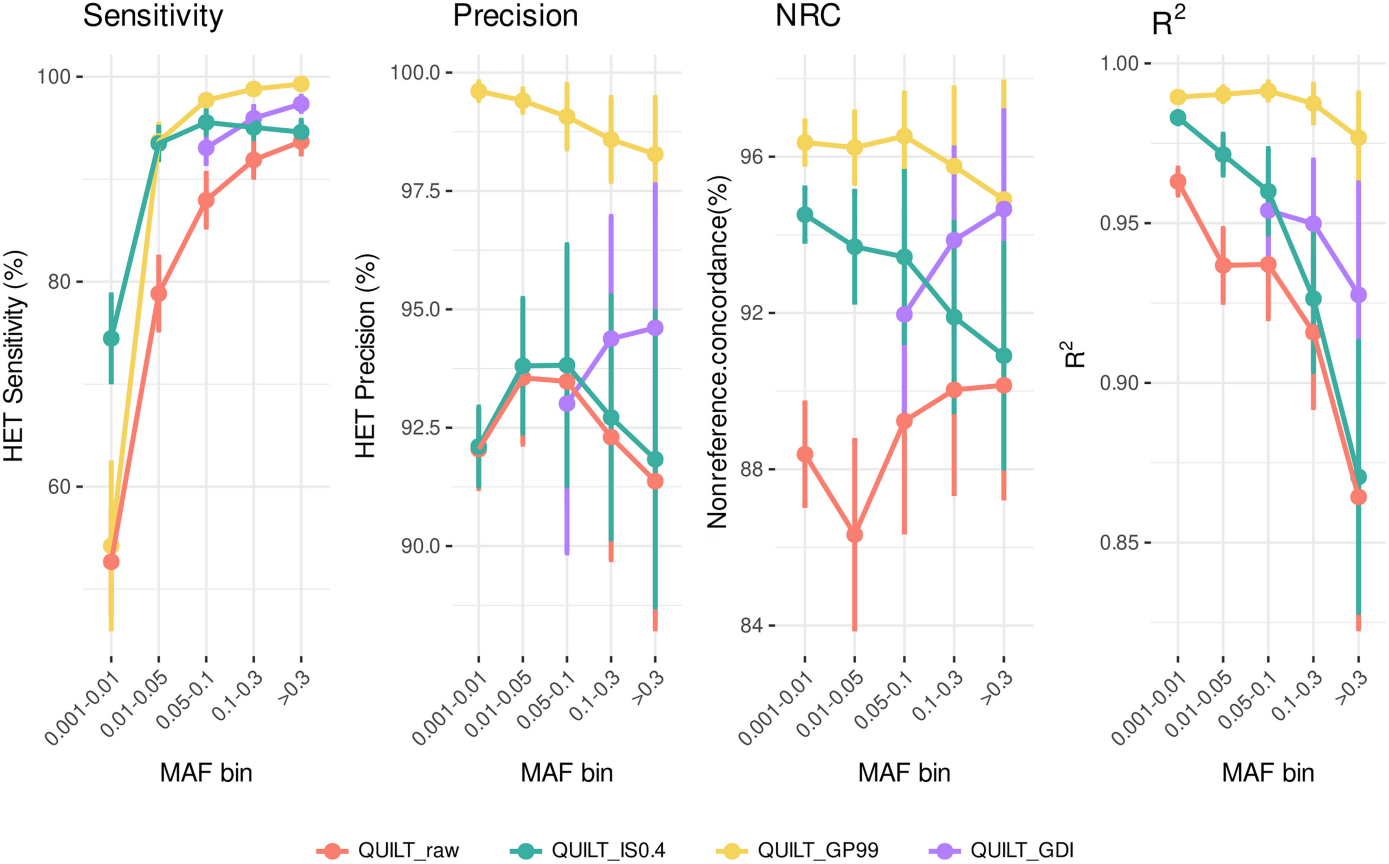
Comparative analysis of postimputation filters on QUILT performance by four measures of accuracy. Each panel displays results for different postimputation statistics for those genotypes imputed as heterozygous: Sensitivity (TP/[TP+FN]), Precision (TP/[TP+FP]), Nonreference concordance (NRC = 1−[Err + Era + Eaa]/[Err + Era + Eaa + Mra + Maa], where Err, Era, and Eaa are the counts of the mismatches for the homozygous reference, heterozygous and homozygous alternative genotypes, and Mra and Maa are the counts of the matches at the heterozygous and homozygous alternative genotypes, and the dosage r-squared (Pearson's *r*^2^ on dosage values calculated using BCFtools stats). Lines are colored based on different filtering approaches. Results are averaged across the three test samples, with SD bars showing the variability around the mean values (refer to Supplemental Table S2 for detailed information on the values). The lack of QUILT_GDI data points in the 0.001–0.01 and 0.01–0.05 frequency bins reflects our focus on common variants with MAF > 5% in the HRC panel.

We compared the performance of QUILT across different postimputation statistics on the three test samples with high coverage data (mean coverage ∼44×) (Fig. 2). Without any filters (raw results in Fig. 2), QUILT shows the lowest accuracy estimates for each of the statistics we examined.

**Figure 3.**
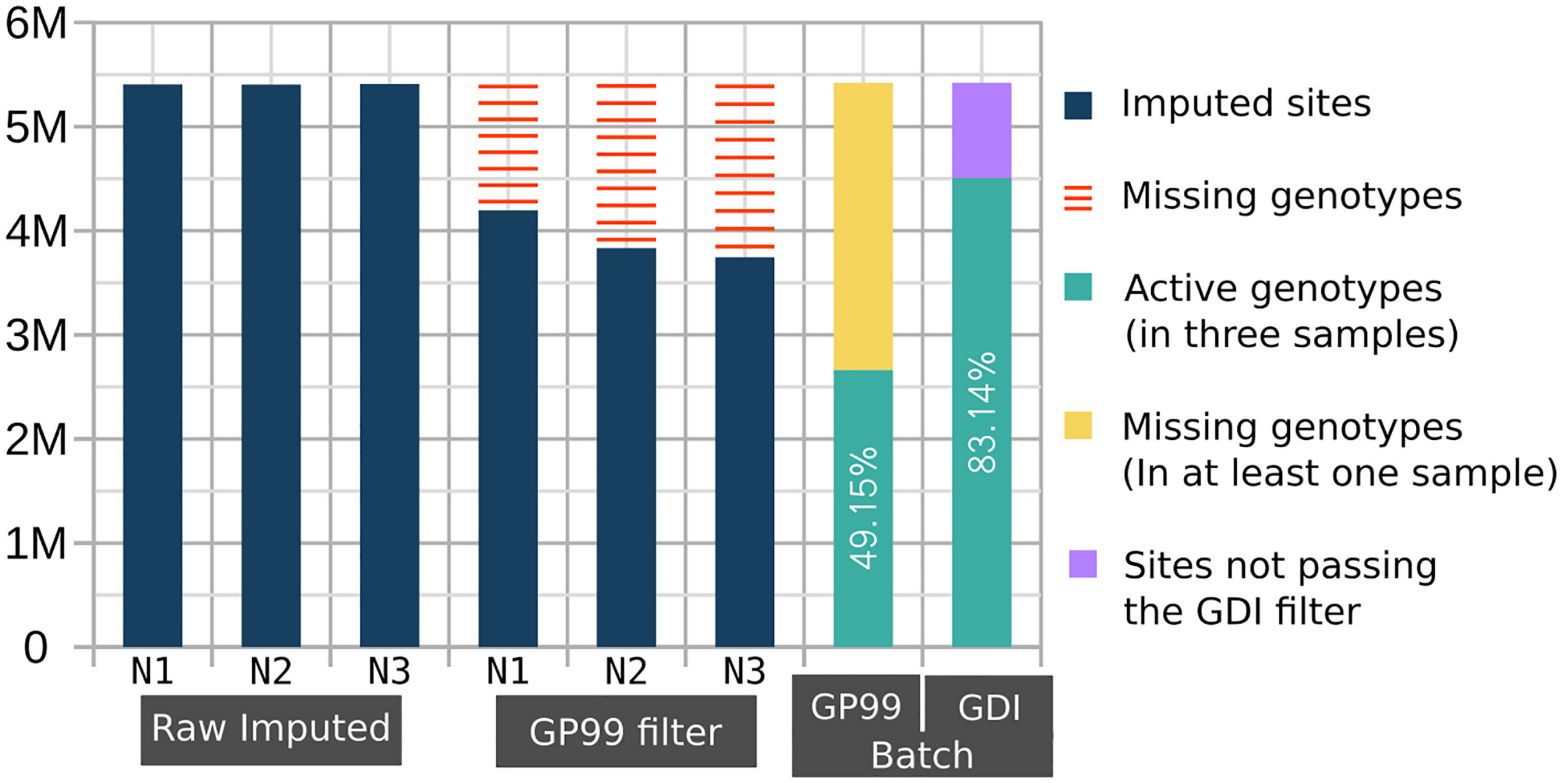
Cumulative missingness of filtered-out variants across multiple imputed genomes. The number of imputed common variants (MAFHRC > 0.05) is displayed in millions on the *y*-axis for each of the three imputed NIPS samples (N1, N2, and N3). The raw imputed data of each individual had 5.4 M variants. Applying GP < 0.99 filter (GP99 filter) on each individual separately results in a variable number of missing variants (red bars). The GP99 and GDI Batch bars to the *right* show the batch effect of cumulative missingness when applying the respective filters. The green bar shows the number of retained sites after removing variants with GP < 0.99 (yellow) in at least one sample or variant sites not passing the GDI filter (lilac).

A notable improvement over unfiltered results was observed when applying INFO score filtering alone (green line in Fig. 2). Because the use of INFO score filtering with QUILT had not been previously explored, we applied the method from Liu et al. (2018), originally designed for STITCH (Davies et al. 2016), to evaluate INFO scores with QUILT. The most notable comparison is between the GDI filtering approach and the GP-based filtering (max(GP) ≥ 0.99) (yellow vs. purple line in Fig. 2). It is evident that both filtering approaches resulted in an improvement compared to the raw data in all the statistics. Our tests confirmed that applying a GP filter at the individual level enhances data quality. Conversely, the GDI filter shows a smaller improvement but is crucial for retaining the majority of variant sites in large batches of data, thereby avoiding the introduction of any missing data, as shown below in case of PCA analyses. Indeed, the GP filtering approach can be effectively used only on a single genome or a few genomes, because in larger batches, most, if not all, variants would have missing genotype calls introduced by the GP filter in at least some individuals. To illustrate the difference on a small example, Figure 3 shows results from a batch of three individual NIPS genomes: the GDI filter retains 83% of variants, whereas the GP filter, applied cumulatively, retains only 49.2% of total sites. These differences increase with larger sample sizes. In other words, Figure 3 illustrates on a small sample that GP filtering at the individual level leads to massive cumulative missingness when the data is pooled. In contrast, the GDI approach maintains a higher proportion of nonmissing variants across the batch, avoiding these issues.

**Figure 4.**
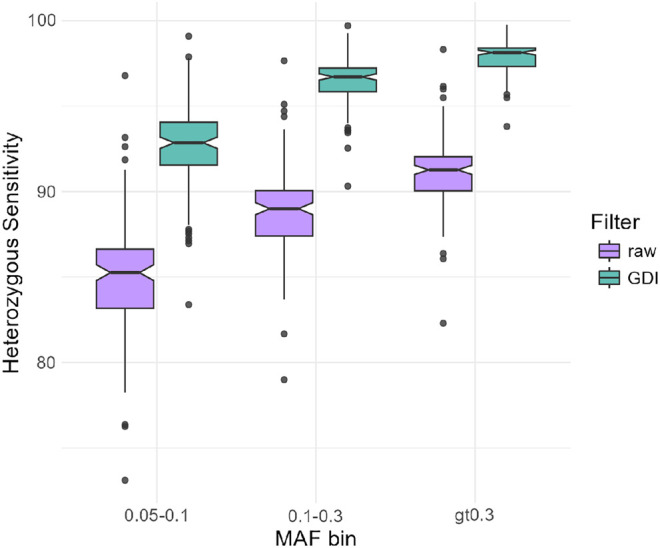
Box plots per MAF showing the heterozygous genotype imputation sensitivity before (raw) and after GDI filters. Data are derived from 138 nonpregnant healthy individuals sequenced at 0.1–0.2× coverage and genotyped, in parallel, on Illumina Infinium Global Screening Array-24 v3.0 for ∼700,000 SNPs. The array-based genotype calls were considered as truth.

To validate the effect of the GDI filtering approach on imputation accuracy in an independent data set, we used LPS and high-quality genotype data from 138 nonpregnant healthy controls that were not part of the GDI filter development. Figure 4 summarizes the results of 138 individuals, showing that, for all MAF bins >0.05, the GDI filtered LPS data had higher heterozygous sensitivity than the raw data. Furthermore, the GDI filtered data scored better in other accuracy statistics as well, such as heterozygous specificity and the nonreference concordance (Supplemental Table S5). Metrics from these additional samples validated our observations from the three NIPS samples. Moreover, these results demonstrate that the GDI filter is robust when applied to other data sets.

#### GDI application

In light of the success of the GDI filtering approach in minimizing the presence of low-quality variants across the largest possible subset of samples, the 28,512 NIPS samples that passed initial quality checks and removal of related samples were further processed using this method. We removed an additional 1158 outliers based on the observed distribution of low-quality variants (LQV scores, defined by the proportion of sites not passing the thresholds set for GP values, DS values, and the INFO score provided by QUILT) (Supplemental Fig. S6). Notably, the average coverage for these 1158 samples is approximately 0.09×, confirming that the LQV metric effectively identified low-coverage samples. These samples were classified as outliers based on their high LQV levels prior to the application of GDI (Supplemental Fig. S11).

Furthermore, the distribution of SeqFF values revealed that samples removed due to the LQV filter exhibited a higher median SeqFF compared to those retained for further analyses (Supplemental Fig. S7). Specifically, the median SeqFF for LQV samples was 10.1, whereas for retained samples, it was 8.99 (Wilcoxon rank-sum test *P*-value = 4.147 × 10^−13^). This confirmed the negative effect of SeqFF on imputation accuracy.

#### Evaluation of GDI effectiveness across coverage levels

To evaluate the effectiveness of the GDI thresholds (INFO, GP, and DS) across different sequencing coverage levels, we grouped the samples into coverage bins and assessed the difference in data quality (LQV) before and after GDI application (Supplemental Fig. S8). The analysis revealed a consistent positive impact on accuracy of the GDI method across all coverage bins, with the most pronounced effect observed at lower coverage levels. For bins in the range ≥0.1–<0.2×, the median indicates that the differences between pre- and post-GDI are more uniformly distributed. However, the internal variability is quite high, with some samples showing much larger improvements (outliers), which likely contributes to the observed overall uniformity in this bin. A similar trend is observed in the ≥0.2 to <0.3× bin, where the differences remain generally uniform but with no extreme outliers. In the ≥0.3 bins, the median indicates that the differences between pre- and post-GDI are generally modest or small. It is important to note that, within each coverage bin, especially for those with coverage ranges of ≥0.1 to <0.2× and ≥0.2 to <0.3×, the large number of samples results in significant variability in the individual sample coverage. This variability may influence the observed results and should be considered when interpreting the data.

#### PCA analyses

The final set of 27,354 samples was used alongside 498 samples from the Genome of the Netherlands (GoNL) (The Genome of the Netherlands Consortium 2014), 3643 samples from the Project MinE (Project MinE ALS Sequencing Consortium 2018), and 2495 samples from the 1000 Genomes Project (The 1000 Genomes Project Consortium 2015) to visually explore the distribution of genetic ancestry in the imputed data with the PCA and UMAP approach, aiming to assess the performance of downstream population genetic analysis methods and to test whether imputation is causing batch effects in the data.

We applied UMAP on 20 PCs estimated from the data, as this approach has been shown to enable the detection of regional clusters at higher resolution than the examination of only the first two PCs (Diaz-Papkovich et al. 2021). Without the GDI filter (raw imputed data), a clear batch effect can be observed in UMAP (Supplemental Fig. S9A); as expected, as UMAP tends to allocate more space to the most represented group (Diaz-Papkovich et al. 2021), LPS samples occupy most of the space, but no clear structure in the data can be observed. A similar result is observed when plotting a UMAP after applying a filter on the INFO score (<0.4) (Supplemental Fig. S5); the plot still shows a strong batch effect, similar to that seen with the raw data in Supplemental Figure S9. This suggests that, although filtering based on the INFO score improves various postimputation statistics, it does not effectively remove the batch effect. However, applying a GDI filter and removing variants with low quality in more than 30% of the samples (Supplemental Fig. S9B) effectively helps to remove this bias and reorganize the data more clearly. In particular, we can see that the larger group predominantly composed of LPS samples (bigger gray cloud in Supplemental Fig. S9A) is merged after filtering with the smaller separate cluster including individuals from the 1000 Genomes Project (GBR and CEU), most of the MinE data set, and the GoNL group. Furthermore, part of the LPS samples is clearly clustering with non-European groups. This batch effect is also detectable in a milder form in the plot of PC1 and PC2 (Supplemental Fig. S9C), where the distribution of the Belgian NIPS samples appears not completely aligned with the distribution of the other data sets of individuals with similar ancestry (MinE and GoNL).

As a general observation, the removal of variants with low quality scores led to the elimination of ∼1.9 million variants, reducing the overall data set of common variants to 3,355,663 variants. Following this removal, an average reduction of 54.1% was observed in the LQV scores compared to prefilter values (Supplemental Fig. S6). Upon removal of the low-quality variants, the imputed LPS data appears to include less individuals outside the triangular structure of the PC plot (compare Supplemental Fig. S9, C and D).

#### Genetic ancestry and population structure in Belgian NIPS data

**Figure 5.**
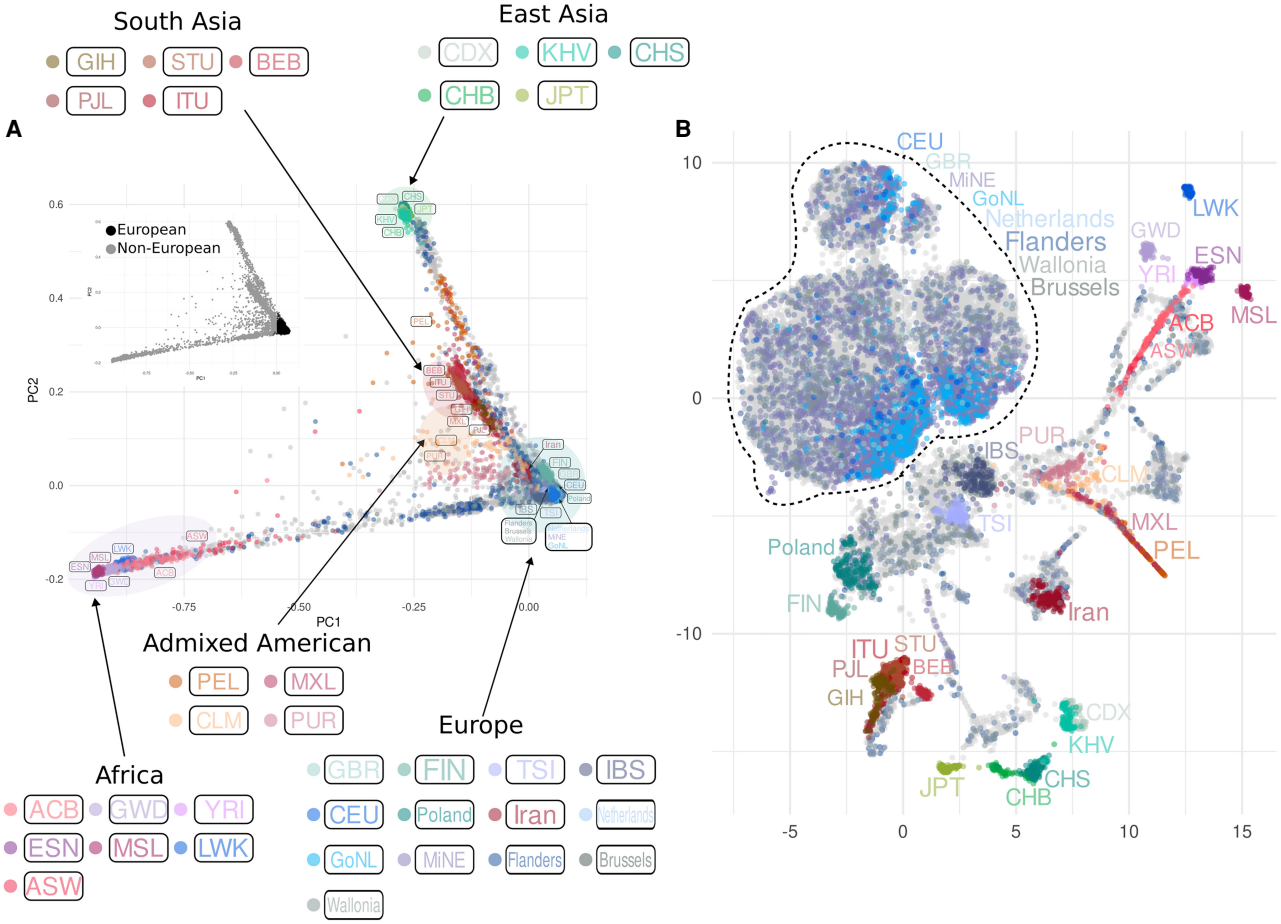
Principal component analysis of imputed NIPS data in the context of regional and global reference populations. (*A*) The plot of PC1 and PC2. The smaller PCA plot highlights European and non-European ancestries. (*B*) UMAP plot of the first 20 PCs. Both plots display the distribution of 27,354 imputed NIPS samples (gray dots in the background) together with samples from the 1000 Genomes Project, the GoNL, and the MinE data sets, after applying a GDI filter removing variants with low quality in more than 30% of the imputed NIPS samples. Population labels are displayed next to the group they represent (samples from Iran and Poland are part of the Belgian data), with colors of the labels matching the colors of the corresponding samples. Unlabeled imputed samples are depicted as gray dots in the background. The dotted line includes the group represented by samples from the GoNL, the MinE, the 1000 Genomes Project (CEU and GBR only), unlabeled NIPS samples, and NIPS individuals labeled as Flanders, Wallonia, Brussels, and the Netherlands.

As genetic ancestry is an important confounding factor for many medical and population genetic analysis tools, it is important to know how sensitive methods that cluster individuals by their ancestry are to imputation quality. When focusing on the results of the first two PCs (Fig. 5A), we first confirmed that imputed NIPS samples exhibit a wide distribution in the context of global reference data: most of the samples are primarily clustered along the European edge of the plot, representing 89.17% of the imputed data, and the remaining 10.83% showed dispersion towards the African and the East Asian edges of the plot.

These results from PCA were also confirmed with a supervised admixture, where 87.3% of the imputed NIPS samples had a European ancestry proportion of ≥90%. Within the subset of NIPS data where country of origin was known, 165 individuals from Iran formed a cluster right below the European edge in the PCA, which becomes more distinct in the UMAP conversion (Fig. 5B). Similarly, after applying the GDI filter, within the European subset a cluster of 131 individuals from Poland becomes more clearly pronounced (Fig. 5B; Supplemental Fig. S9). The majority of NIPS individuals whose birthplace was identified as being in Belgium mapped together with MinE and GoNL individuals on PC plots (Supplemental Fig. S10), and between IBS, CEU, and GBR populations of the 1000 Genomes Project data, consistent with previous observations (Van Den Eynden et al. 2018).

### PGS for height

**Figure 6.**
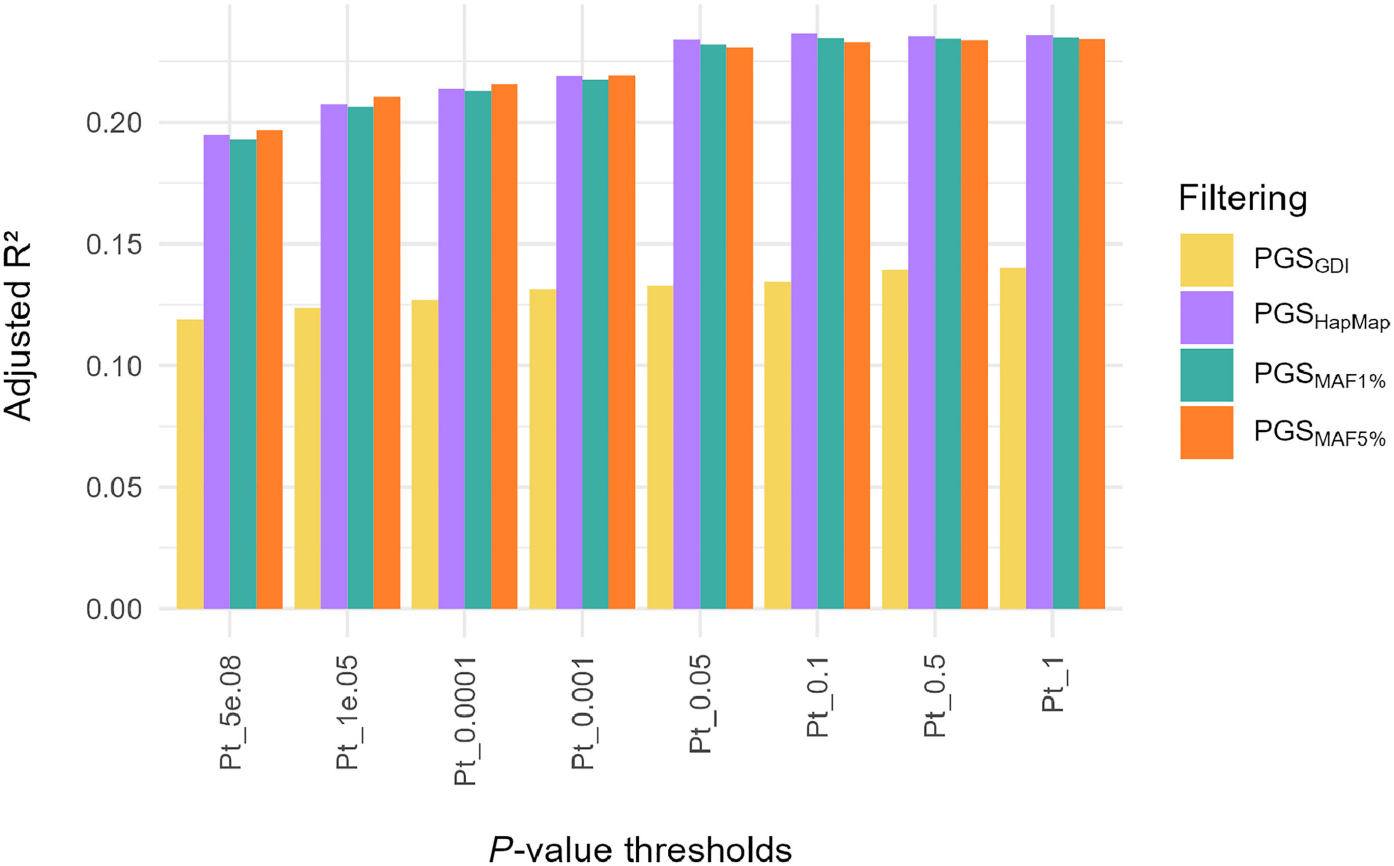
Phenotypic variance in height explained by the PGS per *P*-value threshold and for different filtering approaches. The overall best performing PGS in terms of variance explained is PGSHapMap at *P*-value threshold (Pt) 0.1 with an *r*² of 0.237 (beta = 0.03, *P*-value = 2.83 × 10^−114^).

For 1911 imputed NIPS samples, polygenic scores for height were calculated at different *P*-value thresholds (Pt) using four approaches: (i) data filtered by GDI and MAF ≥ 5%; (ii) data filtered by MAF ≥ 5% only; (iii) data filtered by MAF ≥ 1%; and (iv) data limited to HapMap SNPs. These are referred to as PGS_GDI_, PGS_MAF5%_, PGS_MAF1%_, and PGS_HapMap_, respectively. When performing linear regression, where the height of an individual is predicted by PGS, PGS_GDI_ consistently had a lower variance explained for all Pt values than the other PGSs (Fig. 6), whereas the three other PGSs mostly had similar values (Supplemental Table S4). Overall, the best PGS model was PGS_HapMap_, with a variance explained of 0.237 at Pt = 0.1, including 62,059 SNPs. However, it is worth noting that the variance explained for PGS_HapMap_, PGS_MAF5%_, and PGS_MAF1%_ doesn't change much from Pt = 0.05 onward, indicating that the addition of SNPs with *P*-value > 0.05 does not have much added value towards prediction. We also calculated the Spearman's correlation coefficient (*r*) of the correlation between genotypic (i.e., the PGS) and phenotypic height. For PGS_HapMap_, PGS_MAF5%_, and PGS_MAF1%_, the correlation was the same—*r* = 0.46. This is in contrast with PGS_GDI_ where *r* = 0.36. Together, these findings indicate that filtering the imputed NIPS data with the GDI filter does not improve, at least in case of height, PGS performance in terms of either variance explained or correlation with phenotypic height.

## Discussion

In this study, we explored the potential of genotype imputation of large cohorts of low-pass (0.1×–0.3×) sequence data from Belgium, for the population genetic study of genetic ancestry and PGS estimation for medical risk stratification. In tests performed on an individual level, we found that imputation can be achieved with comparable quality using QUILT (Davies et al. 2021) and GLIMPSE2 (Rubinacci et al. 2023). When using ultralow coverage (<0.2×) sequences without quality filters, imputation accuracy appeared, however, to be too low for applications, such as PCA. Our tests confirmed that imputed genotypes from 0.1× to 0.2× sequence data generated with QUILT and GLIMPSE2 without further quality filters exhibit heterozygote sensitivity <0.95. For downstream applications that require higher imputation accuracy, an alternative approach of applying quality filters, such as GP, separately on each individual leads to high (>0.98) individual sensitivity scores but also introduces cumulative missingness when working on batches of data. As a solution, we introduced batch level filtering approach GDI that optimizes the balance imputation accuracy and the retention of the largest possible subset of samples and variants. A similar result could not be achieved by filtering solely based on INFO score (<0.4), as this approach did not resolve the batch effect observed in the UMAP. However, our results confirmed the validity of using INFO score filtering as a foundation for improving data quality, which is why we adopted this metric as the first step in our GDI filtering strategy. Although we showed that the GDI approach was effective in removing batch effects caused by low imputation accuracy in low coverage cohorts seen in PC analyses, the GDI approach did not improve the PGS prediction of height. We observed negative correlation between fetal fraction and sensitivity across the range of different coverage values (Supplemental Fig. S3), suggesting that, although higher coverage ensures higher imputation accuracy, this relationship may be compromised in cases of high fetal fraction.

The best PGS for height was PGS_HapMap_ with a variance explained of 0.237 and a correlation with phenotypic height of 0.46 (Fig. 6). PGS_MAF5%_ and PGS_MAF1%_ had similar results. In contrast, with PGS_GDI_ only 14% of phenotypic variance of height could be explained (Supplemental Table S4). A possible reason for this finding could be that there were less SNPs available to build the PGS compared to data that was only MAF 1% or MAF 5% filtered. However, the SNP count alone cannot fully explain the observed difference in variance explained and correlation. This was demonstrated by PGS_HapMap_, which had the best performance even though there were less SNPs in the initial data set than the GDI filtered data. On the other hand, it is important to note that, even though in the HapMap filtered data set there are less SNPs than in the GDI data set, these SNPs have been chosen to capture most of the common variability in the human genome (The International HapMap Consortium 2005). Common SNPs, which are predominantly the signals detected by GWAS, are consequently the ones used for PGS. Together, this could explain why the PGS_GDI_ is not performing as well as the other filtered data sets. Of note, we only calculated a PGS for the complex trait height, which is highly polygenic—or even omnigenic—in nature (Boyle et al. 2017; Yengo et al. 2022). Hence, these results could differ for other traits or diseases that have a different genetic architecture. One commonly observed cause of the drop of variance explained in PGS models is the mismatch between genetic ancestry of the test cohort and the GWAS cohort on which the GPS model is based. However, we cannot think of a reason why the GDI filter would remove specifically variants that are informative for height PGS in Europeans. Furthermore, the imputation reference panels we used were chosen to maximize the success of genotype imputation in a cohort of predominantly European ancestry.

When selecting the most appropriate reference panel for imputation, we tried to ensure that it would be representative of all major genetic ancestry components of the sampled population. As the samples were predominantly from Flemish hospitals, we expected the majority of our cohort to have European ancestry, with the addition of ∼10% of Asian or African ancestry. We compared the performance of a global reference panel of the 1000 Genomes Project (The 1000 Genomes Project Consortium 2015) and the Haplotype Reference Consortium panel (The Haplotype Reference Consortium 2016) which includes the former and is enriched for individuals with predominantly European ancestry. Given the larger number of individual sequences and the number of variants the HRC panel provided, we achieved a higher imputation accuracy and found a smaller number of variants that had to be filtered out (Table 1; Supplemental Tables S2, S3). We observed the greatest improvement of accuracy for variants with MAF 0.01 to 0.05, which may have importance for downstream applications that consider variants in this low frequency spectrum. The imputation accuracy of rare variants (MAF < 0.01) remained too low with both panels and filter settings for any downstream purposes examined in this study.

One possible strategy to improve imputation quality at the batch level is to apply filtering by summary quality measures calculated for the entire batch. INFO score is the only such measure available in the QUILT output. However, with large cohorts that are constantly being supplemented with new data, such as the 32,769 individuals examined here, it can be preferable for practical reasons to carry out imputations in smaller batches and to merge them in the end for a common set of variants. Only 131,908 variants (2.4% of common variants) had INFO score below the 0.4 threshold in all the batches of NIPS data we studied, which offered only a minor improvement of quality over the option of using no filters. Furthermore, we observed that incorrectly imputed variants included many with high INFO score, a subset of which had a wrongly imputed genotype whereas their GP and DS values pointed consistently to the genotype called from the high coverage data. This motivated us to carry out systematic revisions of conflicts between the GT and GP values, which could be characteristic only for ultralow coverage data, and to focus our search for optimal solutions in the GDI design on the combination of INFO score, GP, and DS filters. We acknowledge that, in ideal circumstances, sequencing to the minimum depth of 0.5× should be encouraged to reach sufficient accuracy for downstream applications that would not require further filtering (Sousa de Mota et al. 2023). However, in case of data sets that have already been generated in the past and/or where it is challenging, if not impossible, to generate more data, the GDI approach may offer leverages for data that otherwise cannot be used for genomic scale genotype analyses. Application of the GDI filter after imputation enabled us to achieve imputation accuracy in the large batch of 32,769 individuals on par with, although less superior than, that achieved with the GP filter applied at individual level (Fig. 2). However, the advantage represented by the GDI approach over the GP filter at a batch level was significant as it allowed us to prevent the cumulative introduction of missing data by each individual, keeping the majority (83%) of the imputed common variants for downstream analyses. Furthermore, when we assessed the effectiveness of GDI across varying coverage levels, we observed that the method generally improved data quality in all coverage categories. The greatest improvements were noted in samples with lower coverage, whereas higher coverage bins displayed more variability in the degree of improvement. This variability may reflect factors such as differences in sample characteristics and coverage distribution within each bin. These results demonstrate the broad applicability of GDI, while suggesting that fine-tuning of the thresholds may be needed for data sets with higher coverage.

The study by Sousa de Mota et al. (2023) showed that individuals imputed from 0.1× to 0.25× coverage were correctly placed in the expected continental clusters in PCA, while showing at the same time significant deviations from the precise placement within those clusters. Our results confirm this: when using imputed genotype data without filters as the input, we find that the majority of individuals map in the PC1 and PC2 plot to the context of the reference sources from Belgium and the Netherlands, as expected, whereas in the higher resolution UMAP analyses of P1-20 data, we find that the unfiltered data cluster separately from the others (Supplemental Fig. S9). With our final settings of the GDI filter, which retained 3,355,663 out of 5,421,789 common variants (61.9%), we were able to remove the separate clustering of NIPS data as a batch. Regarding the determination of the cutoff for variant removal in the GDI filter, we leveraged visual exploration of the sample distribution on a UMAP plot after a series of filtering steps, with increments of 10% of LQV sites being removed at each step, to guide our decision-making. However, in scenarios where such visual exploration is not feasible, the fundamental principle guiding this approach is to strike a balance between being overly conservative and excessively aggressive in variant removal, avoiding the loss of a substantial number of variants. Although it might seem arbitrary, custom-oriented handling of this step is essential, recognizing that, in principle, each combination of a data set and a downstream data analysis method can be unique and necessitate tailored consideration.

As we sought a solution for using 0.1×–0.3× sequence data in ancestry analyses, we developed the specific combination GDI filters to be optimized for the performance of high resolution PCA on Belgian NIPS data, and the same parameter settings we used may not offer the most optimal solution in other settings. We expect, however, that when appropriate reference panels are available (e.g., for other European cohorts with similar coverage data), the GDI filtering following QUILT (or GLIMPSE) imputation can be a useful approach for genetic ancestry analyses with PCA. Other downstream analyses, including those focused on PGS, can, most likely, benefit from individual filter optimizations that are different, either more conservative or stringent, from those described and explored here. In cases where increasing the sequence coverage is not an option, a generic strategy that may prove effective should involve exploration of the distribution of the number of low-quality variants against the number of samples in which they fail, aiding in defining the most appropriate cutoffs and optimal reduction in LQV scores. A further point worth stressing here is that, because the imputation of heterozygous sites is always the most challenging, it is highly likely that such sites are the ones with lower GP and DS values and therefore do not pass the GDI filter. As a consequence, more stringent filters can lead to a reduction of heterozygosity in the sample.

Applying a genome-wide SNP array of around 300,000 sites on 189 individual samples from Belgium, Van den Eynden et al. (2018) showed the typical European genetic constitution of the Belgian population. Our exploratory analyses of the downstream effects of imputation quality filters on principal component analysis have facilitated the study of the genetic ancestry of 32,769 pregnant individuals recently collected from a hospital in present-day Flanders at the genomic scale for more than 3 million common variants. This has provided a higher resolution view on the genetic ancestry of present-day Flanders and has enabled better quantification of the extent of recent migration. The majority of the samples group within regional clusters of Belgian and Dutch individuals from the MinE and GoNL projects, whereas a minor subset clusters with other European subgroups and individuals with non-European genetic background. These results are not surprising given Belgium's long history of immigration and integration. Historically, Belgium is known to have been a nation characterized by persistent immigration, a phenomenon that has undoubtedly contributed significantly to shaping its demographic landscape. Indeed, this continuous demographic evolution has increasingly enriched what is now Belgium's population, which to date welcomes individuals from different corners of the world (Martiniello 2013). Notably, 10.83% of the imputed samples mapped outside the main European cluster in PCA (Fig. 4A), which is similar to a recent estimate, according to which 11% of the population living in the Flemish Region were born outside the EU. Given that our samples come primarily from Flemish hospitals, they represent the demographic diversity of individuals residing in Belgium, including not only those of Belgian origin but also a broader spectrum. As a result, our findings offer an accurate portrayal of the diverse population found in contemporary Belgium, truly reflecting the cosmopolitan nature of the country and providing a realistic snapshot of its current demographics.

## Methods

A concise description of the materials and methods used in this study is provided below. For a comprehensive and detailed version of all protocols and procedures, please refer to the Supplemental Methods.

### Data collection

Peripheral blood samples were collected from 32,769 pregnant individuals undergoing NIPS and from 140 nonpregnant healthy controls, with ethics approval from KU Leuven and University Hospitals Leuven (S63253, S66621, S66450). cfDNA was extracted from plasma and sequenced at low-pass coverage (median ∼0.15×) using Illumina platforms, following protocols previously described (Bayindir et al. 2015). Sequence data were aligned to GRCh38 (hg38), and standard QC, deduplication, and sorting procedures were applied. For the 140 controls, high-quality genotyping was also performed for benchmarking imputation accuracy.

### Imputation tool testing

To select the optimal imputation tool, we evaluated GLIMPSE, GLIMPSE2, QUILT, and a dual-step strategy combining either with Beagle. Imputation accuracy was benchmarked using three low-pass NIPS samples (∼0.16×) with known fetal fractions and corresponding high-coverage whole-blood genomes. All tools were applied using the HRC reference panel, and accuracy was assessed per genotype class and MAF bin using metrics such as sensitivity and dosage *r*².

### Reference panel setup

All imputation strategies used either the HRC or 1000 Genomes Project reference panels, both of which were preprocessed using standard liftOver and variant filtering procedures.

### Imputation with QUILT

Genome-wide genotype imputation of the 32,769 samples was performed using QUILT with the HRC panel. Samples were processed in parallel batches using a custom pipeline (Fig. 1A), with chromosomes divided into 5-Mb windows. Post-imputation filtering retained 5.42 million variants with MAF > 5% in the HRC panel for downstream analysis.

### Postimputation filters

#### Removal of duplicates and related individuals

Related individuals were identified and removed based on kinship coefficients estimated with IBIS, resulting in the exclusion of 4257 samples.

#### Filter on variants at batch level

To enhance the quality of the imputed data, we implemented a ﬁltering strategy we called GDI (Fig. 1B) that combines filters on genotype-related metrics, including the posterior genotype probability, alternate allele dosage (DS), and the INFO score provided by QUILT. The INFO score provides a quantitative measure of the certainty associated with genotype imputation based on the distribution and uniformity of genotype posteriors; a low score indicates a flat, noninformative distribution, whereas a score near 1 suggests concentrated, confident genotypes. The DS field represents the expected number of alternate alleles for a given genotype. For diploid genotypes, the DS values should range from 0 to 2. Ideally, a DS equaling 0 means that both alleles are the reference allele (0/0), a DS equaling 1 means that one allele is the reference allele and the other is the alternate allele (0/1), and a DS equaling 2 means that both alleles are the alternate allele (1/1). However, it is possible for the DS values to assume intermediate values between 0 and 2 for diploid genotypes. This can happen when there is uncertainty in the genotype call, such as when the genotype posterior probabilities in the GP field are not clearly in favor of one genotype over another. In this light, the dosage value provides an indication of how well the genotype is supported by imputation. As for the GP field, it represents a measure of how likely each possible genotype at a site is after imputation, with values closer to 1 indicating a higher likelihood and greater confidence in the accuracy of the predicted genotypes following imputation.

When imputing multiple samples with QUILT, the INFO score associated with each variant represents a consensus score. Due to imputing our samples in separate batches, different INFO scores were obtained for the same variants. To address this variability, we aggregated all INFO scores for the same variants across multiple imputed batches. Variants consistently tagged with an INFO score <0.4 across all batches totaled 131,908 and were consequently identified for removal. The initial step of the GDI strategy involved generating a file containing the GT, GP, and DS fields for each variant and individual. Subsequently, variants tagged for removal from the INFO score screening were excluded from further analysis in subsequent steps. For each sample, a list of variants to be removed is generated, determined by a GP < 0.99 and DS ranges defined by the GT field (DS > 0.1 for GT 0/0, DS < 1.8 for GT 1/1, and 0.8 > DS > 1.01 for GT 0/1). The DS thresholds are determined based on theoretical expectations and practical considerations, rather than empirical testing. These values account for minor variations and provide a margin of safety in variant classification. By setting these thresholds, we aimed to ensure accurate classification despite potential fluctuations in DS values. The proportion of variants earmarked for removal defines the fraction of low-quality variants (LQV score) for each sample, and an observation of the distribution based on LQV scores was conducted to establish a cutoff. Samples with an LQV score exceeding 40% were identified as outliers, leading to the removal of a total of 1158 samples from subsequent analyses.

The initial step concludes by generating, for each sample, a list reporting sites that do not pass the DS and GP filters. In the second step of the GDI strategy, these lists are used to gather information on the quality of sites for all individuals (except those identified as outliers in the first GDI step and those flagged as duplicates or relatives by IBIS). Ultimately, variants exhibiting low quality in more than 30% of the samples were excluded, totaling 1,933,956 variants. The choice of this cutoff was defined by the visual observation of the distribution of the imputed data in the context of a UMAP plot based on 20 PCs including also samples from the 1kGP (lifted over to build GRCh38 from the HRC panel), the MinE (lifted over to build GRCh38 with Crossmap), and the GoNL data sets (lifted over to build GRCh38 with Crossmap). The filtered data set comprised 27,354 samples and 3,355,663 variants, reflecting a reduction of ∼11% in the sample count and about 38% of the total variants.

The average reduction of the proportion of LQV sites was calculated by taking the percentage difference for each sample, followed by calculating the mean and standard deviation of these differences. The standard deviation of 6.2% suggests that variations between individual samples and the mean are relatively consistent.

After applying the GDI filter (Supplemental Fig. S12), we observed that 86.4% of the variants had successfully passed all the filters, 4.3% failed the DS filter, 5.1% failed the GP filter, and 4.2% failed both the DS and the GP filters. However, when focusing only on the GP values, we can see that 90.7% of the variants remaining in the data set have a GP ≥ 0.99, 3.06% have a GP between 0.9 and 0.99, and only 6.24% have a GP < 0.9. Overall, the majority of retained variants in our data set exhibit a GP ≥ 0.99, highlighting a significant presence of reliable genetic variations. This underscores the effectiveness of the GDI filter in ensuring data integrity and enhancing overall variant quality.

Additionally, when observing the correlation between the LQV scores and the coverage of each sample, we observed that, as the coverage increases, the LQV score tends to decrease. This suggests that samples with higher coverage tend to have fewer low-quality variants, indicating a potential association between higher coverage and improved quality of imputed variants. Before applying the filter, we calculated the correlation between the coverage and the LQV scores for each sample and observed a strongly negative value (Spearman's correlation of −0.75). This indicates that higher coverage values correspond to lower LQV scores. In practice, this suggests that samples with higher coverage will be associated with a lower proportion of low-quality variants. After the filter, we observed a reduction in the correlation value (Spearman's correlation of −0.66), indicating that, despite the reduction in LQV scores, the negative correlation with the coverage persists. In addition, the observed mean LQV score decreased from 0.28 to 0.13 after the application of the filter, indicating a consistent reduction in the proportion of low-quality variants among the samples. Furthermore, the standard deviation changed from ±0.0524 to ±0.0396, indicating an increase of the consistency and homogeneity of the postfilter data. Overall, these results show that the application of the filter affected the distribution of low-quality variants among the samples, contributing to increased uniformity of LQV values and a persistent correlation between coverage and LQV scores, albeit slightly attenuated.

#### Comparison with other filtering strategies

To assess the effectiveness of our GDI method compared to other filtering strategies, we employed the three test samples and compared the results using different postimputation statistics for the raw imputed data, the GDI strategy, and a direct filter on max(GP) ≥ 0.99. Additionally, we conducted a cross-method comparison by applying imputation through QUILT, GLIMPSE, and GLIMPSE2. Imputation performances were observed over different allele frequency ranges (MAF bins).

### Principal components analysis and UMAP

Population structure was assessed through PCA and UMAP embedding using LD-pruned variants across imputed NIPS samples and external references (1kGP, MinE, GoNL).

### PGS calculation

To calculate the polygenic scores, we used the effect sizes provided by the genome-wide association study for height by Yengo et al. (2022). Duplicate, ambiguous, and multiallelic SNPs were removed, and a MAF filter of 1% was applied.

Out of the 32,769 NIPS samples, only 2698 had a reported height available. After excluding samples because of relatedness or being an outlier (see above), non-European samples were excluded as well. Samples were included as European based on a supervised admixture analysis, where samples with a proportion of ≥0.95 European ancestry were retained. Based on these criteria, 1911 samples were included for the PGS calculation.

PRSice-2 (Choi and O'Reilly 2019) was used for the calculation of PGS for height for a range of predefined *P*-value thresholds (Pt) (5 × 10^−8^, 1 × 10^−5^, 1 × 10^−4^, 1 × 10^−3^, 0.05, 0.1, 0.5, 1) with the following clumping parameters: distance to both ends from the index SNP = 250 kb, *r*² = 0.1, and *P*-value threshold = 1. Scores were calculated for individuals from the non-Finnish European 1000 Genomes Project (1KG-NFE), as well as for 1911 NIPS samples, using the same SNPs per Pt as selected for the 1KG-NFE scores. Four different filtering methods were used to calculate the scores: (1) 1KG-NFE were MAF 5% filtered, then the selected SNPs were used to calculate scores per Pt in the GDI filtered NIPS (PGSGDI); (2) 1KG-NFE were MAF 5% filtered, then the selected SNPs were used to calculate scores per Pt in MAF 5% filtered NIPS (PGSMAF5%); (3) 1KG-NFE were MAF 1% filtered, then the selected SNPs were used to calculate scores per Pt in MAF 1% filtered NIPS (PGSMAF1%); and (4) an overlap was taken between 1KG-NFE and HapMap SNPs, then the selected SNPs were used to calculate scores per Pt in NIPS (PGSHapMap). The 1KG-NFE data set contained 6,064,728 SNPs, 8,743,364 SNPs, or 1,116,280 SNPs after filtering for MAF 5%, MAF 1%, or HapMap SNPs, respectively.

After calculation of the raw scores, the first 10 principal components (PCs) were regressed out of the scores. Subsequently, the PC-corrected scores were then standardized against the PC-corrected scores from the 1KG-NFE individuals. Specifically, the scores of each individual were standardized by subtracting the mean score of individuals from the 1KG-NFE group from their own scores and then dividing the resulting value by the standard deviation of the scores within 1KG-NFE. When we use the term PGS, we refer to the standardized PC-corrected scores.

### Declaration of generative AI in the writing process

During the preparation of this manuscript, the authors used ChatGPT to improve the language and readability of the text. After using this tool, the authors reviewed and edited the content as needed and take full responsibility for the final version of the manuscript.

### Software availability

All source code and custom scripts used in this study are available at GitHub (https://github.com/SABiagini/GDI and https://github.com/SABiagini/PostImputationStats), and as Supplemental Code.

## Data access

The NIPS sequence data used in this study are available under restricted and controlled access, in compliance with the GDPR. The genomic data are locally stored at UZ Leuven, and access can be obtained with permission from the local UZ Leuven data access committee (DAC: https://www.uzleuven.be/en/dac, dac@uzleuven.be; cc: joris.vermeesch@uzleuven.be). The access process is further described in https://www.uzleuven.be/en/data-access-committee-dac/external-applicant-requests-access-ku-leuven-researchers-dataset. Sequence data of the 140 healthy nonpregnant individuals, used as controls for imputation accuracy, is available through the European Nucleotide Archive (EGA; https://ega-archive.org/) under accession number EGAS50000001114.

## Supplemental Material

Supplement 1

Supplement 2

Supplement 3

Supplement 4

Supplement 5

Supplement 6

Supplement 7

Supplement 8
