# Supplementary figures and images for: Genotype imputation from low-coverage data for medical and population genetic analyses
Source: Genome Res. 2025 Sep;35(9):1929–41. doi: 10.1101/gr.280175.124 (PMC12400947; doi:10.1101/gr.280175.124)

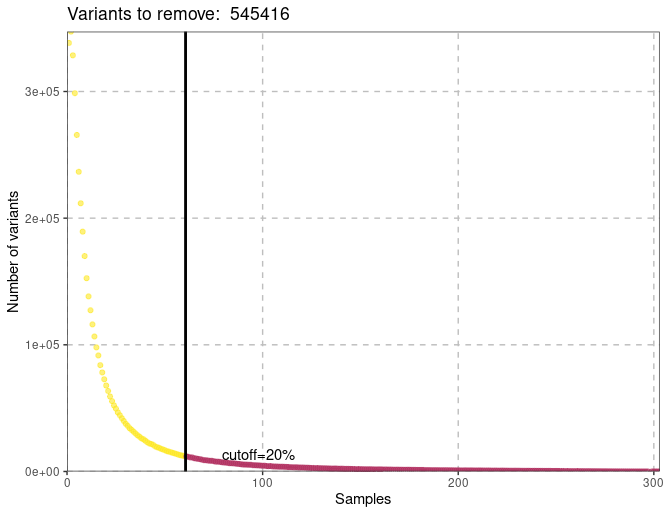

Supplement: Supplement 1 [file Supplemental_Code.zip › Supplemental_Code/GDI-main/Figures/cutoff_20perc.png]

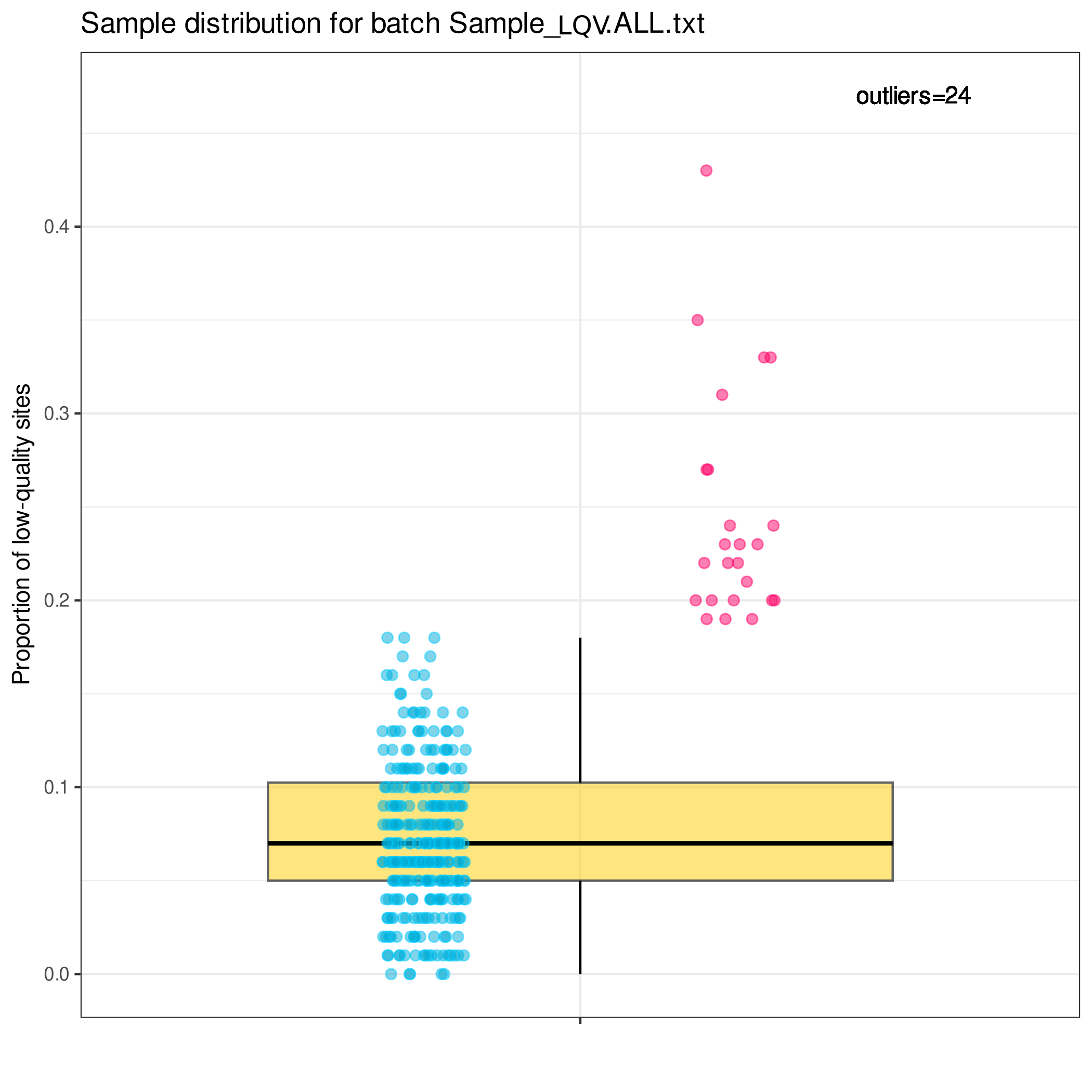

Supplement: Supplement 1 [file Supplemental_Code.zip › Supplemental_Code/GDI-main/Figures/LQV_preGDI.txt.png]

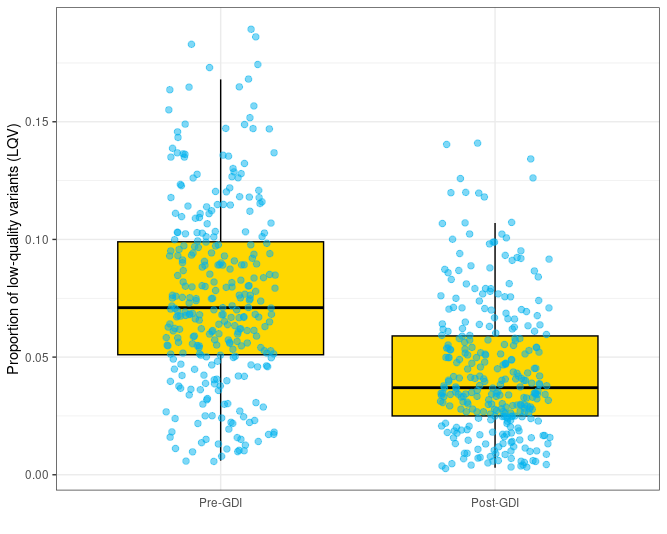

Supplement: Supplement 1 [file Supplemental_Code.zip › Supplemental_Code/GDI-main/Figures/pre_post_GDI.png]
