# Supplementary material for: Genotype imputation from low-coverage data for medical and population genetic analyses
Source: Genome Res. 2025 Sep;35(9):1929–41. doi: 10.1101/gr.280175.124 (PMC12400947; doi:10.1101/gr.280175.124)
Supplement: Supplement 2 [file Supplemental_Methods.pdf]

MATERIALS AND METHODS

Data collection

This study was approved by the Ethics Committee Research of KU Leuven and the University Hospitals Leuven (study protocols S63253, S66621, and S66450).

LPS data was generated with methods described elsewhere (Bayindir et al. 2015). Briefly, peripheral blood samples from pregnant individuals were collected in cfDNA collection tubes (Roche Diagnostics, Switzerland or Streck, USA). Plasma was separated by the standard dual centrifugation method and cfDNA extracted with the QIAamp Circulating Nucleic Acid Kit (Qiagen Benelux B.V., Venlo, Netherlands) according to the manufacturer's guidelines. Sequencing libraries were prepared with TruSeq ChIP-seq Library Preparation Kit (Illumina, San Diego, CA, USA) according to the manufacturer's recommendations with modifications described elsewhere (Bayindir et al. 2015). Samples were indexed during library preparation and sequencing was performed on the HiSeq 2500 (Illumina) in fast mode producing 50 bp single-end reads. Raw reads were aligned to the human reference genome hg38 using the Burrows-Wheeler aligner (v0.7.17). Duplicate reads were marked and removed using Picard (v2.18.23). Bams were sorted and indexed with SAMtools (v1.9).

Additional peripheral blood samples were collected from 140 healthy non-pregnant individuals. Plasma was separated by the standard dual centrifugation method and cfDNA extracted with the QIAamp Circulating Nucleic Acid Kit (Qiagen Benelux B.V., Venlo, Netherlands) or Maxwell HT ccfDNA kit (Promega; automated procedure) according to the manufacturer's guidelines. Sequencing libraries were prepared with TruSeq ChIP-seq Library Preparation Kit (Illumina, San Diego, CA, USA) or KAPA HyperPrep kit (Roche Diagnostics) according to the manufacturer's recommendations with modifications described elsewhere (Bayindir et al. 2015). Samples were indexed during library preparation and sequencing was performed on

the HiSeq 2500 (Illumina) in fast mode producing 50 bp single-end reads or NovaSeq 6000 (Illumina) 50 bp paired-end. In addition, these samples were genotyped from genomic DNA extracted separately on Illumina Infinium Global Screening Array-24 v3.0 (custom array with add-on content EstChip2-GSAv3-MD, ~700,000 SNPs) for the estimation of imputation accuracy (Figure 4). Following QC on both sequence and genotype data, the data for 138 individuals was used in the downstream analyses.

The fetal fraction of the NIPS data, for pregnancies with both male and female fetus, was determined with the SeqFF method, which is based on multivariate models trained on autosomal regional read counts (Kim et al. 2015) and in case of male pregnancies by measuring the relative abundance of autosomal and X Chromosome reads at the 50 kb bin level (Rava et al. 2014).

Average autosomal sequence depth of the 32,769 NIPS genomes was calculated from their BAM files using mosdepth (Pedersen and Quinlan 2018). Figure S11 illustrates the coverage distribution of the 32,769 imputed NIPS samples, showing distinct patterns across the different categories. The 27,354 retained samples exhibit a broad distribution of coverage values, ranging from 0.08× to 0.99×, with a median of approximately 0.15×. This wide range of coverage values further supports the representativeness of the dataset, as it includes a diverse set of samples reflecting varying levels of coverage. In contrast, the 1,158 outliers identified through LQV filtering are concentrated below the first quartile (Q1, ~0.13×), with coverage values primarily between 0 and 0.1×. The 4,257 kinship-based outliers, on the other hand, are distributed between 0.08× and 0.46×, with notable variation in coverage both below and above the median. These findings underscore the efficiency of LQV filtering in targeting low-coverage samples, while also highlighting the variable nature of kinship-based exclusions across the coverage spectrum.

Imputation tool testing

The imputation tool selection was conducted using three low-coverage NIPS test samples (average coverage ~0.16×), each with a different fetal fraction (low=3.4%, intermediate=7.4%, and high=10.3%). To enable direct estimation of accuracy of imputation from NIPS data, we sequenced the DNA extracted from the whole blood of these three donors to high coverage. Sequence depth estimates calculated with mosdepth from the CRAM files were: 29.9×, 70.×x, and 31.7×. After mapping, genotypes from these high coverage sequences were called using GATK v. 4.1.2.0 (Poplin et al. 2017). To determine the most suitable imputation tool, we employed various methods: GLIMPSE, GLIMPSE2, QUILT, and a dual-step imputation strategy combining Beagle with GLIMPSE and GLIMPSE2. For Glimpse, a preliminary variant calling of the samples was performed using GATK's HaplotypeCaller, followed by the standard protocol defined by the Glimpse authors. In the dual-step imputation, imputed data with Glimpse underwent a filtering step, setting to missing genotypes with GP values lower than 0.99 (the BCFtools +tag2tag plugin was employed for this operation setting the option -t 0.01) (Li 2011); upon this filter, data were passed to Beagle for a second round of imputation, following default procedure. Regarding GLIMPSE2, we followed the default protocol using the static binaries provided by the authors, while in the dual-step imputation we repeated the same procedure described for GLIMPSE. As for QUILT, the BAM files of the three samples were directly used as input, following the standard procedures of the imputation tool. For each method employed, imputation was carried out on individual chromosomes, using the Haplotype Reference Consortium (HRC) (the Haplotype Reference Consortium 2016) as reference panel. A custom script was designed to compare imputed and high coverage data using SnpSift (Cingolani et al. 2012) and compute various post-imputation statistics, among which are sensitivity, the number of correctly imputed variants, their proportion, and dosage r-squared values (Pearson's r^2 on dosage values calculated using BCFtools stats). These calculations were performed separately for each genotype (homozygous for the reference allele, homozygous for the alternate allele, and heterozygous) and for different minor allele frequency (MAF) bins (0.001-0.01, 0.01-0.05, 0.05-0.1, 0.1-0.3, >0.3, and >0.5). The true

positive rate (TPR or sensitivity) has been calculated as the ratio of true positive sites (TP) to the sum of true positives and false negatives (TP+FN) and defines the probability that an actual positive will test positive.

Reference panel setup

For each imputation strategy employed, the HRC panel served as the reference dataset. Firstly, the panel was lifted over to the GRCh38 human genome build using Crossmap (Zhao et al. 2014) and filtered to exclude sites with a Minor Allele Count lower than 5, extra contigs (variants from regions with ambiguous chromosomal assignments), positions with ambiguous nucleotides (IUPAC codes like K, R, W, Y, and B), duplicates based on position (same position but different rs ID), and REF/ALT swaps (inverted REF/ALT alleles respect to the 1000 Genomes Project b38 data). Following this filtering procedure, an average loss of approximately 130,500 variants was noted across the 22 autosomes in comparison to the original HRC data. The final panel comprised 27,165 individuals and 36,258,911 variants. With this procedure we aimed to enhance the reliability of subsequent analyses and maintain the overall quality of the reference panel. This approach prioritizes precision and data integrity, aligning with the goal of achieving accurate imputation outcomes. For the 1000 Genomes reference panel, the same filtering steps were used as for the HRC panel. The final 1000 Genomes reference panel contained 2,504 samples and 32,140,179 variants.

Imputation with QUILT

To optimize computational efficiency, the reference panel underwent preprocessing using the *QUILT_prepare_reference.R* script, and the output was saved for subsequent independent executions. In our pipeline (Figure 1, panel A), a total of 32.769 samples were organized into batches of 1250 samples each, where possible. For each batch, 30 separate jobs were initiated for the imputation of the 22 autosomal chromosomes, each handling a variable

number of windows simultaneously, depending on the chromosome size (those chromosomes with a higher number of windows were split in two parts, and this explains why the total number of jobs per batch is 30 and not 22).

The Worker framework (https://github.com/gjbex/worker) was employed to manage the parallelization of multiple tasks. Each chromosome was partitioned into 5 Mb non-overlapping windows, totaling 567 windows, with a 250 kbp buffer set using the QUILT --buffer flag. Imputation for each window occurred in parallel for 5 samples at a time (setting the flag --nCores=5). For instance, Chromosome 22 was divided into 8 non-overlapping windows of 5 Mb each, a worker was launched for a batch of 1250 samples, and the 8 windows were concurrently managed, imputing 5 samples at a time. To expedite processing, 4 batches were simultaneously launched, handling a total of 5000 samples concurrently. In terms of resources, the number of allocated cores varied based on the number of windows to impute, with 4-5 cores dedicated to each window and a 4GB memory allocation per window. More than one node was never needed. On average, processing a batch of 1250 samples took between 5 and 6 days.

Following imputation, the windows were concatenated using BCFtools concat. In exceptional cases, imputation anomalies, represented by incorrect multiallelic calls for certain genotypes (e.g., cases like 3|0, 4|1, 0|2), were addressed by setting them as missing. GTs were forced to match the most likely GP values for those imputed sites where the assigned GT deviated from the most likely GP. This adjustment was performed using the BCFtools +tag2tag plugin, resulting in the loss of the phasing obtained with QUILT. Eventually, all batches were merged with BCFtools and, for downstream analyses, the resulting file was filtered to retain only variants with a minor allele frequency greater than 5% in the HRC panel (MAF_{HRC} >5%), resulting in a total of 5,421,789 variants out of the 36,258,911 million imputed ones.

Post imputation filters

Removal of duplicates and related individuals

Kinship coefficient calculation was conducted using IBIS v.1.20.9 on a PLINK file containing a total of 32,769 samples and 5,421,789 variants. This process involved extracting pairs of individuals who shared extended unphased LSAI segments, following the criteria of one shared segment of >5 cM (–min_L 5). Eventually, 4,257 samples were selected for removal.

Filter on variants at batch level

To enhance the quality of the imputed data, we implemented a filtering strategy we called GDI (Figure 1, panel B) that combines filters on genotype-related metrics, including the posterior genotype probability (GP), alternate allele dosage (DS) and the INFO score provided by QUILT. The INFO score provides a quantitative measure of the certainty associated with genotype imputation based on the distribution and uniformity of genotype posteriors; a low score indicates a flat, non-informative distribution, while a score near 1 suggests concentrated, confident genotypes. The DS field represents the expected number of alternate alleles for a given genotype. For diploid genotypes, the DS values should range from 0 to 2. Ideally, a DS equal 0 means that both alleles are the reference allele (0/0), a DS equal 1 means that one allele is the reference allele and the other is the alternate allele (0/1), and a DS equal 2 means that both alleles are the alternate allele (1/1). However, it is possible for the DS values to assume intermediate values between 0 and 2 for diploid genotypes. This can happen when there is uncertainty in the genotype call, such as when the genotype posterior probabilities in the GP field are not clearly in favor of one genotype over another. In this light, the dosage value provides an indication of how well the genotype is supported by imputation. As for the GP field, it represents a measure of how likely each possible genotype at a site is after imputation, with values closer to 1 indicating a higher likelihood and greater confidence in the accuracy of the predicted genotypes following imputation.

When imputing multiple samples with QUILT, the INFO score associated with each variant represents a consensus score. Due to imputing our samples in separate batches, different INFO scores were obtained for the same variants. To address this variability, we aggregated all INFO scores for the same variants across multiple imputed batches. Variants consistently tagged with an INFO score <0.4 across all batches totaled 131,908 and were consequently identified for removal. The initial step of the GDI strategy involved generating a file containing the GT, GP, and DS fields for each variant and individual. Subsequently, variants tagged for removal from the INFO score screening were excluded from further analysis in subsequent steps. For each sample, a list of variants to be removed is generated, determined by a GP <0.99 and DS ranges defined by the GT field (DS > 0.1 for GT 0/0, DS < 1.8 for GT 1/1, and 0.8 > DS > 1.01 for GT 0/1). The DS thresholds are determined based on theoretical expectations and practical considerations, rather than empirical testing. These values account for minor variations and provide a margin of safety in variant classification. By setting these thresholds, we aim to ensure accurate classification despite potential fluctuations in DS values. The proportion of variants earmarked for removal defines the fraction of low-quality variants (LQV score) for each sample, and an observation of the distribution based on LQV scores was conducted to establish a cutoff. Samples with an LQV score exceeding 40% were identified as outliers, leading to the removal of a total of 1.158 samples from subsequent analyses.

The initial step concludes by generating, for each sample, a list reporting sites that do not pass the DS and GP filters. In the second step of the GDI strategy, these lists are used to gather information on the quality of sites for all individuals (except those identified as outliers in the first GDI step and those flagged as duplicates or relatives by IBIS). Ultimately, variants exhibiting low quality in more than 30% of the samples were excluded, totaling 1,933,956 variants. The choice of this cutoff was defined by the visual observation of the distribution of the imputed data in the context of a UMAP plot based on 20 PCs including also samples from

the 1KGP (lifted over to build GRCh38 from the HRC panel), the MinE (lifted over to build GRCh38 with Crossmap), and the GoNL datasets (lifted over to build GRCh38 with Crossmap). The filtered dataset comprised 27,354 samples and 3,355,663 variants, reflecting a reduction of approximately 11% in the sample count and about 38% of the total variants.

The average reduction of the proportion of LQV sites was calculated by taking the percentage difference for each sample, followed by calculating the mean and standard deviation of these differences. The standard deviation of 6.2% suggests that variations between individual samples and the mean are relatively consistent.

After applying the GDI filter (Figure S12), we observed that 86.4% of the variants had successfully passed all the filters, 4.3% failed the DS filter, 5.1% failed the GP filter, and 4.2% failed both the DS and the GP filters. However, when focusing only on the GP values, we can see that the 90.7% of the variants remaining in the dataset have a GP ≥0.99, the 3.06% have a GP between 0.9 and 0.99, and only a 6.24% have a GP < 0.9. Overall, the majority of retained variants in our dataset exhibit a GP ≥0.99, highlighting a significant presence of reliable genetic variations. This underscores the effectiveness of the GDI filter in ensuring data integrity and enhancing overall variant quality.

Additionally, when observing the correlation between the LQV scores and the coverage of each sample, we observed that as the coverage increases, the LQV score tends to decrease. This suggests that samples with higher coverage tend to have fewer low-quality variants, indicating a potential association between higher coverage and improved quality of imputed variants. Before applying the filter, we calculated the correlation between the coverage and

the LQV scores for each sample and observed a strongly negative value (Spearman's correlation is -0.75). This indicates that higher coverage values correspond to lower LQV scores. In practice, this is suggesting that samples with higher coverage will be associated with a lower proportion of low-quality variants. After the filter, we observed a reduction in the correlation value (Spearman's correlation is -0.66) indicating that despite the reduction in LQV scores, the negative correlation with the coverage persists. In addition, the observed mean LQV score decreased from 0.28 to 0.13 after the application of the filter, indicating a consistent reduction in the proportion of low-quality variants among the samples. Furthermore, the standard deviation changed from ±0.0524 to ±0.0396, indicating an increase of the consistency and homogeneity of the post-filter data. Overall, these results show that the application of the filter affected the distribution of low-quality variants among the samples, contributing to increased uniformity of LQV values and a persistent correlation between coverage and LQV scores, albeit slightly attenuated.

Comparison with other filtering strategies

To assess the effectiveness of our GDI method compared to other filtering strategies, we employed the three test samples and compared the results using different post imputation statistics for the raw imputed data, the GDI strategy, and a direct filter on max(GP) ≥ 0.99. Additionally, we conducted a cross-method comparison by applying imputation through QUILT, GLIMPSE, and GLIMPSE2. Imputation performances were observed over different allele frequency ranges (MAF bins).

Principal components analysis and UMAP

Principal component analysis (PCA) was conducted on LD pruned data with r^2=0.5 (144.935 variants) using FlashPCA2 (Abraham, Qiu, and Inouye 2017) , with the parameter for the number of output PCs set to 20 (flag -d 20). Besides the 27,354 imputed NIPS samples, the PCA included 2,495 samples from the 1000 Genomes Project, 3,643 samples from the MinE dataset, and 498 samples from the GoNL dataset. Uniform Manifold Approximation and Projection (UMAP) was performed on 20 PCs using the *umap* function from the *uwot* R package (Melville J 2023), configuring the parameters with a choice of 15 for *n_neighbors* and 0.5 for *min_dist*.

PGS calculation

To calculate the polygenic scores (PGS), we used the effect sizes provided by the genome-wide association study (GWAS) for height by Yengo et al. (Nature 2022). Duplicate, ambiguous and multi-allelic SNPs were removed and a MAF filter of 1% was applied.

Out of the 32,769 NIPS samples, only 2,698 had a reported height available. After excluding samples because of relatedness or being an outlier (see above), non-European samples were excluded as well. Samples were included as European based on a supervised admixture analysis, where samples with a proportion of >= 0.95 European ancestry were retained. Based on these criteria, 1,911 samples were included for the PGS calculation.

PRSice-2 (Choi and O'Reilly 2019) was used for the calculation of PGS for height for a range of predefined p-value thresholds (Pt) (5×10^{-08}, 1×10^{-05}, 1×10^{-04}, 1×10^{-03}, 0.05, 0.1, 0.5, 1) with the following clumping parameters: distance to both ends from the index SNP = 250 kb, r^2 = 0.1 and p-value threshold = 1. Scores were calculated for individuals from the non-Finnish European 1000 Genomes Project (1KG-NFE), as well as for 1,911 NIPS samples, using the same SNPs per Pt as selected for the 1KG-NFE scores. Four different filtering methods were used to calculate the scores: (1) 1KG-NFE were MAF 5% filtered, then the selected SNPs were used to calculated scores per Pt in the GDI filtered NIPS (PGS_{GDI}); (2) 1KG-NFE were

MAF 5% filtered, then the selected SNPs were used to calculate scores per Pt in MAF 5% filtered NIPS ($PGS_{MAF5\%}$); (3) 1KG-NFE were MAF 1% filtered, then the selected SNPs were used to calculate scores per Pt in MAF 1% filtered NIPS ($PGS_{MAF1\%}$); and (4) an overlap was taken between 1KG-NFE and HapMap SNPs, then the selected SNPs were used to calculate scores per Pt in NIPS (PGS_{HapMap}). The 1KG-NFE dataset contained 6,064,728 SNPs, 8,743,364 SNPs, or 1,116,280 SNPs after filtering for MAF 5%, MAF 1% or HapMap SNPs respectively.

After calculation of the raw scores, the first ten principal components (PCs) were regressed out of the scores. Subsequently, the PC-corrected scores were then standardized against the PC-corrected scores from the 1KG-NFE individuals. Specifically, the scores of each individual were standardized by subtracting the mean score of individuals from the 1KG-NFE group from their own scores and then dividing the resulting value by the standard deviation of the scores within 1KG-NFE. When we use the term PGS, we refer to the standardized PC-corrected scores.

Declaration of Generative AI in the Writing Process

During the preparation of this manuscript, the authors used ChatGPT to improve the language and readability of the text. After using this tool, the authors reviewed and edited the content as needed and take full responsibility for the final version of the manuscript.

Software availability

All source code and custom scripts used in this study are available in the GitHub repositories https://github.com/SABiagini/GDI and https://github.com/SABiagini/PostImputationStats.

REFERENCES

Abraham, Gad, Yixuan Qiu, and Michael Inouye. 2017. “FlashPCA2: Principal Component Analysis of Biobank-Scale Genotype Datasets” ed. Oliver Stegle. *Bioinformatics* 33(17): 2776–78. doi:10.1093/bioinformatics/btx299.

Bayindir, Baran, Luc Dehaspe, Nathalie Brison, Paul Brady, Simon Ardui, Molka Kammoun, Lars Van Der Veken, et al. 2015. “Noninvasive Prenatal Testing Using a Novel Analysis Pipeline to Screen for All Autosomal Fetal Aneuploidies Improves Pregnancy Management.” European Journal of Human Genetics 23(10): 1286–93. doi:10.1038/ejhg.2014.282.

Choi, Shing Wan, and Paul F O’Reilly. 2019. “PRSice-2: Polygenic Risk Score Software for Biobank-Scale Data.” GigaScience 8(7): giz082. doi:10.1093/gigascience/giz082.

Cingolani, P., Platts, A., Wang, L.L., Coon, M., Nguyen, T., Wang, L., Land, S.J., Lu, X., Ruden, D.M., 2012. A program for annotating and predicting the effects of single nucleotide polymorphisms, SnpEff: SNPs in the genome of Drosophila melanogaster strain w1118 ; iso-2; iso-3. Fly 6, 80–92. https://doi.org/10.4161/fly.19695

Kim, Sung K., Gregory Hannum, Jennifer Geis, John Tynan, Grant Hogg, Chen Zhao, Taylor J. Jensen, et al. 2015. “Determination of Fetal DNA Fraction from the Plasma of Pregnant Women Using Sequence Read Counts: Determination of Fetal DNA Fraction from the Plasma of Pregnant Women Using Sequence Read Counts.” Prenatal Diagnosis 35(8): 810–15. doi:10.1002/pd.4615.

Li, Heng. 2011. “A Statistical Framework for SNP Calling, Mutation Discovery, Association Mapping and Population Genetical Parameter Estimation from Sequencing Data.” *Bioinformatics* 27(21): 2987–93. doi:10.1093/bioinformatics/btr509.

Melville J. 2023. “Uwot: The Uniform Manifold Approximation and Projection (UMAP) Method for Dimensionality Reduction.”

Pedersen, Brent S, and Aaron R Quinlan. 2018. “Mosdepth: Quick Coverage Calculation for Genomes and Exomes” ed. John Hancock. *Bioinformatics* 34(5): 867–68. doi:10.1093/bioinformatics/btx699.

Poplin, Ryan et al. 2017. *Scaling Accurate Genetic Variant Discovery to Tens of Thousands of Samples*. Genomics. preprint. doi:10.1101/201178.

Rava, Richard P, Anupama Srinivasan, Amy J Sehnert, and Diana W Bianchi. 2014. “Circulating Fetal Cell-Free DNA Fractions Differ in Autosomal Aneuploidies and Monosomy X.” Clinical Chemistry 60(1): 243–50. doi:10.1373/clinchem.2013.207951.

the Haplotype Reference Consortium. 2016. “A Reference Panel of 64.976 Haplotypes for Genotype Imputation.” *Nature Genetics* 48(10): 1279–83. doi:10.1038/ng.3643.

Yengo, Loïc, Sailaja Vedantam, Eirini Marouli, Julia Sidorenko, Eric Bartell, Saori Sakaue, Marielisa Graff, et al. 2022. “A Saturated Map of Common Genetic Variants Associated with Human Height.” Nature 610(7933): 704–12. doi:10.1038/s41586-022-05275-y.

Zhao, Hao et al. 2014. “CrossMap: A Versatile Tool for Coordinate Conversion between Genome Assemblies.” *Bioinformatics* 30(7): 1006–7. doi:10.1093/bioinformatics/btt7
