# Supplementary material for: Genotype imputation from low-coverage data for medical and population genetic analyses
Source: Genome Res. 2025 Sep;35(9):1929–41. doi: 10.1101/gr.280175.124 (PMC12400947; doi:10.1101/gr.280175.124)
Supplement: Supplement 3 [file Supplemental_Figures.pdf]

SUPPLEMENTARY FIGURES

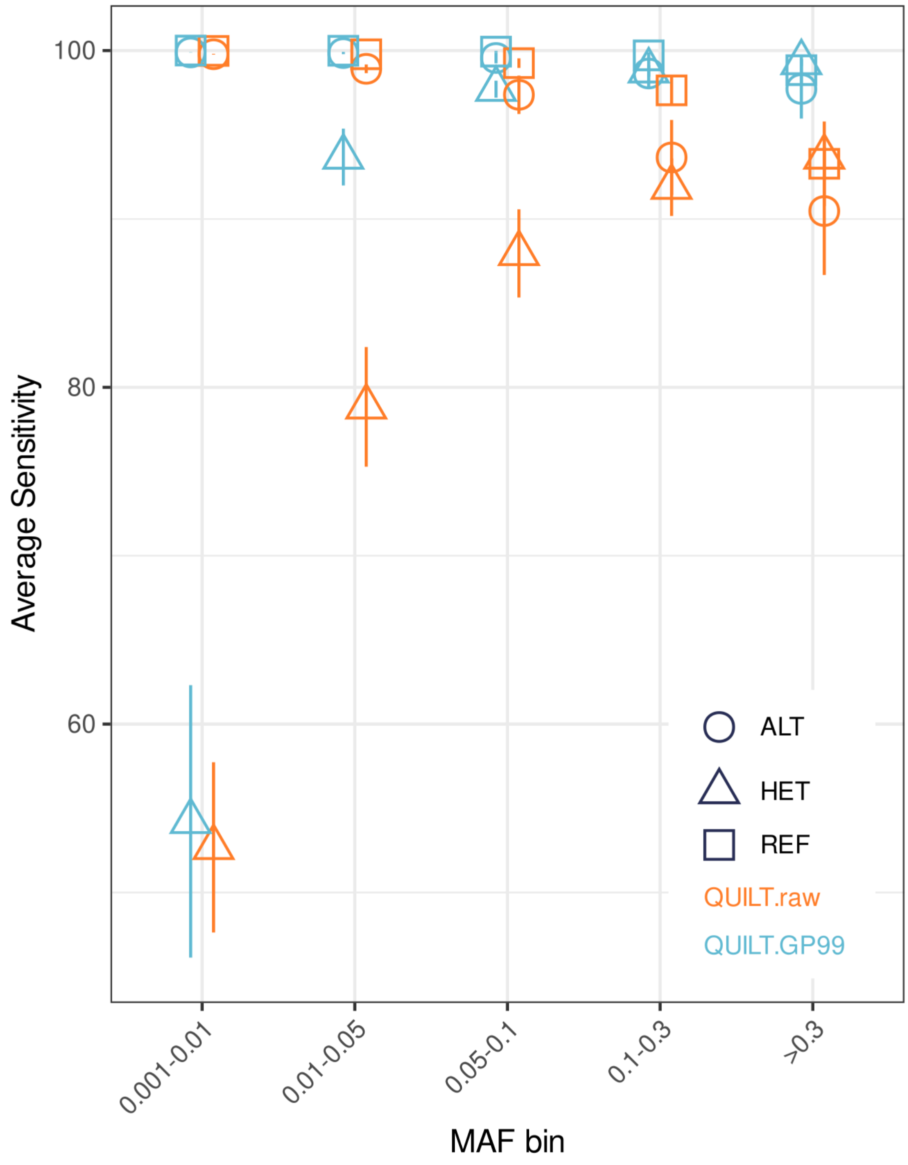

Figure S1 Sensitivity of QUILT across different MAF bins. Average sensitivities of heterozygous genotypes (HET), homozygous genotypes of the reference (REF) and alternative (ALT) alleles were estimated from the data of three individuals whose genotypes were imputed with QUILT from 0.1-0.2x coverage NIPS data and compared to genotypes directly called from the 30x coverage sequence data. Standard deviation (SD) bars are displayed for each average point. For each MAF bin, average values for both raw imputed (orange) and GP-filtered (max(GP) ≥0.99; blue) data are displayed. For further details see Supplementary Table 1.

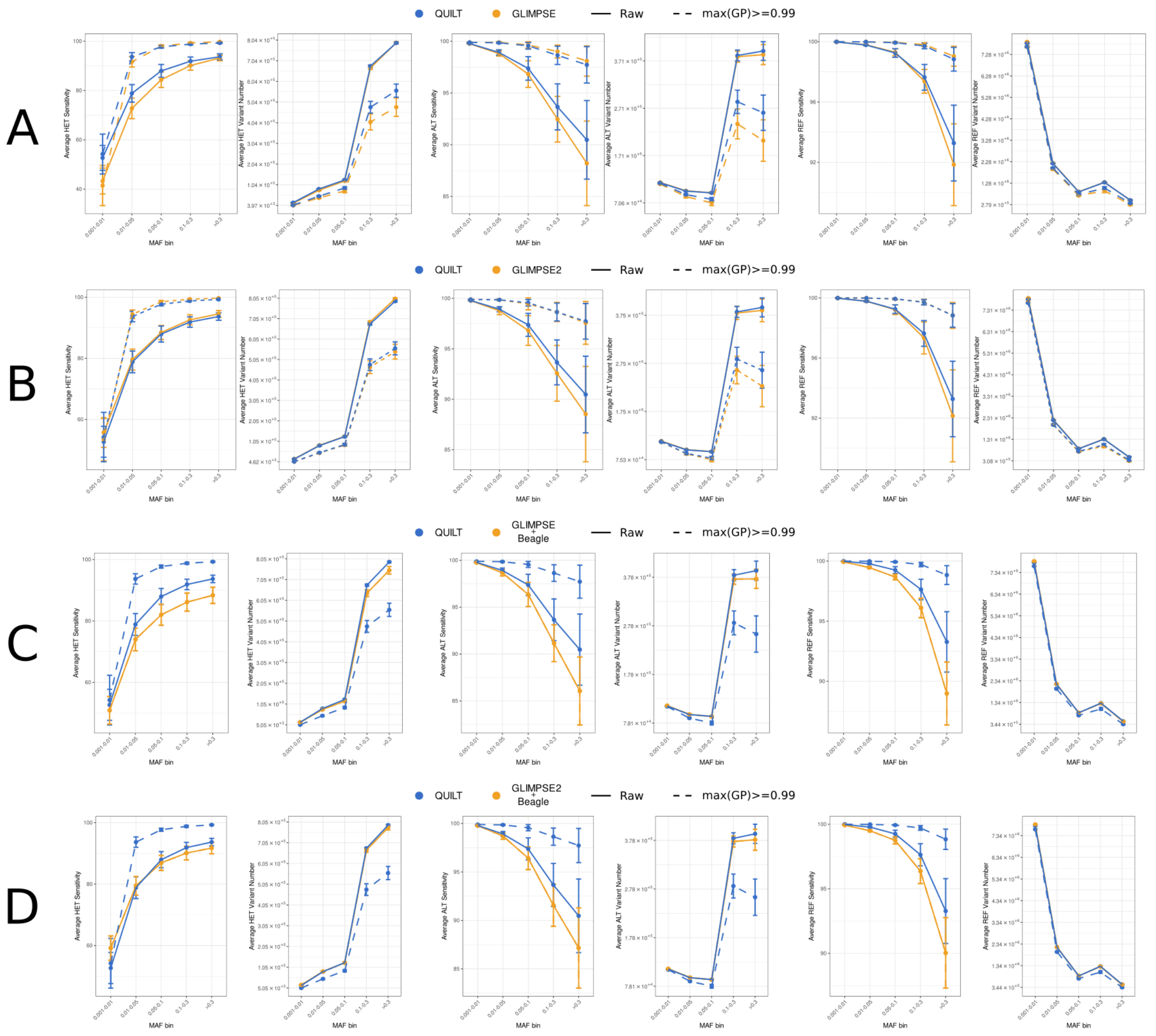

Figure S2 Each panel represents a different comparison between QUILT and other imputation strategies: **A)** QUILT Vs GLIMPSE; **B)** QUILT Vs GLIMPSE2; **C)** QUILT Vs GLIMPSE+Beagle; **D)** QUILT Vs GLIMPSE2+Beagle. Within each panel, we present six separate figures. Three of them detail average sensitivity values calculated across the three test samples for each imputed genotype (REF, ALT, and HET) and for different MAF bins, with standard deviation bars (SD) shown. The remaining three figures display the average number of correctly imputed sites for REF, ALT, and HET genotypes across different MAF bins, also with standard deviation bars (SD) at each average value. Colors distinguish results obtained with QUILT (blue) from those obtained with the compared strategy (yellow). Solid lines represent raw imputed results, while dashed lines represent GP-filtered (max(GP) ≥ 0.99) imputed data. The x-axis of each plot displays the different minor allele frequency bins. For details on the data used for this plot, please refer to Supplementary Table 2.

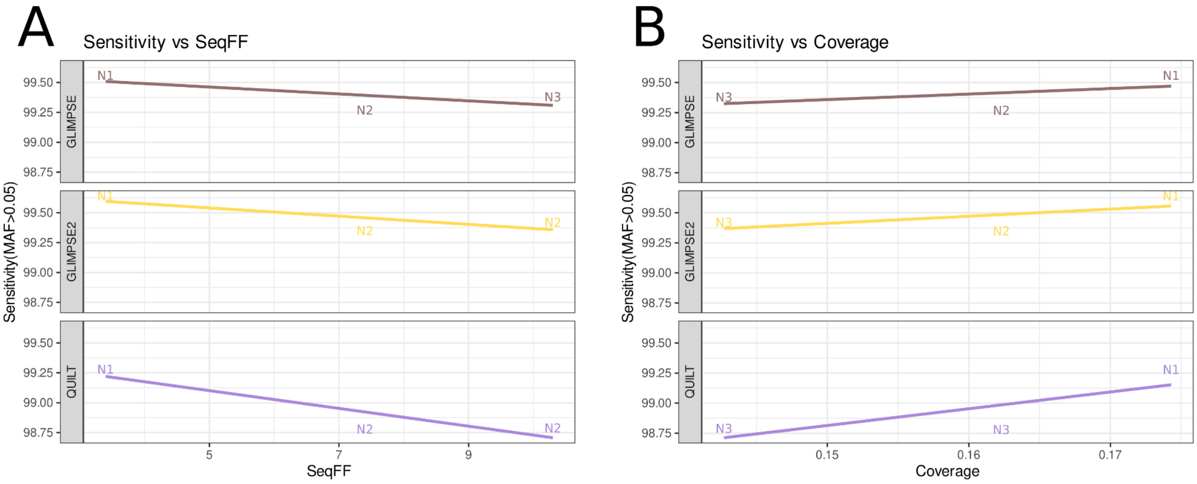

Figure S3 **A)** Sensitivity for common variants (MAF > 0.05) plotted against the fetal fraction (SeqFF). **B)** Sensitivity for common variants (MAF > 0.05) plotted against coverage. The results are presented in separate panels for QUILT, GLIMPSE, and GLIMPSE2, showcasing the performance for the three test samples. Sensitivity values are derived from imputed data filtered by genotype probability with a threshold of max(GP)≥0.99.

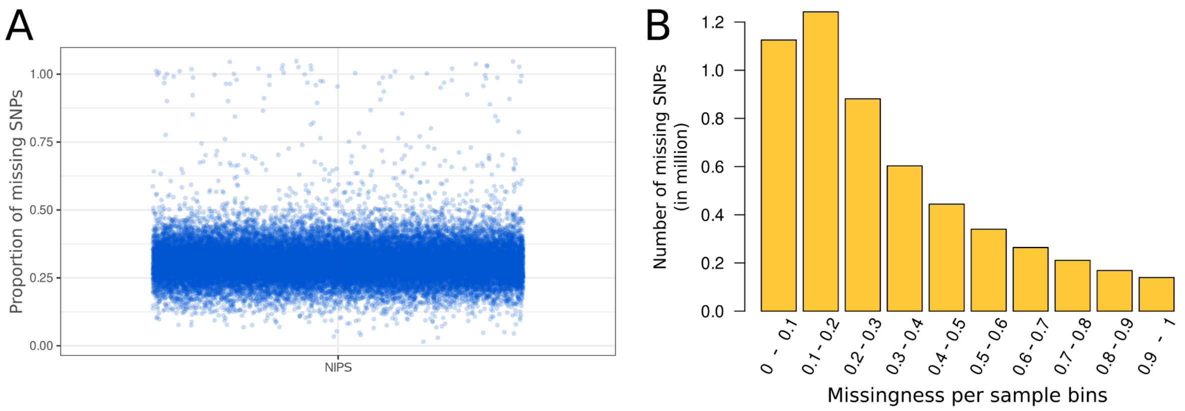

Figure S4 Impact of a GP filter on variant numbers in large batches of imputed data. **A)** Individual missingness after applying the GP filter (max(GP) ≥ 0.99) on 5.4 M common variants in imputed NIPS data. Each point represents an imputed sample, with the Y-axis indicating the proportion of missing SNPs. **B)** Number of variants with missing data in bins of 32.769 NIPS samples, where each bar represents a proportion of samples with missing data and the Y axis displays the number of missing SNPs (in millions).

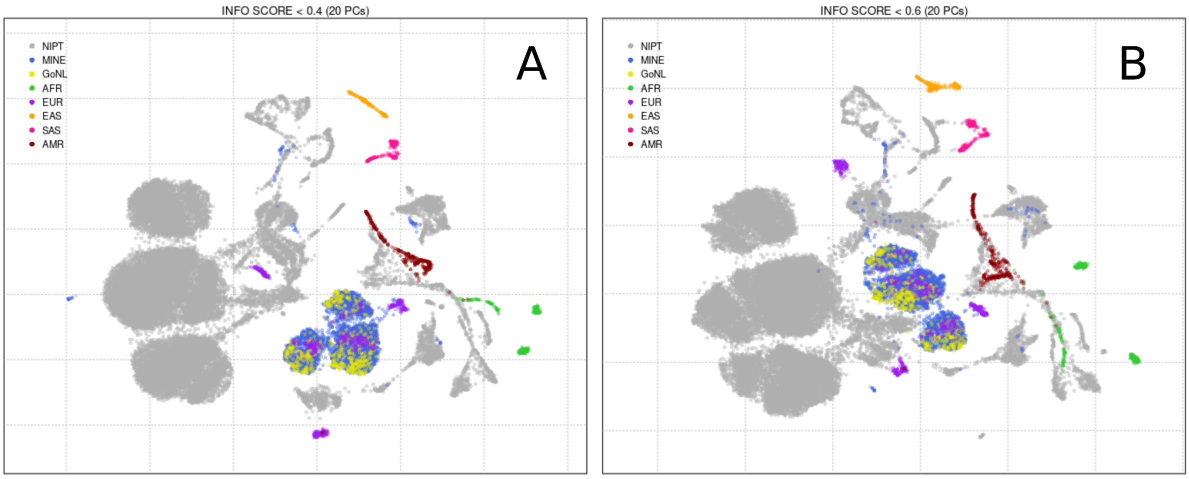

Figure S5 UMAP plots illustrating batch effect after excluding 131,908 imputed variants with INFO score <0.4 (A) and 428,135 imputed variants with INFO score <0.6 (B).

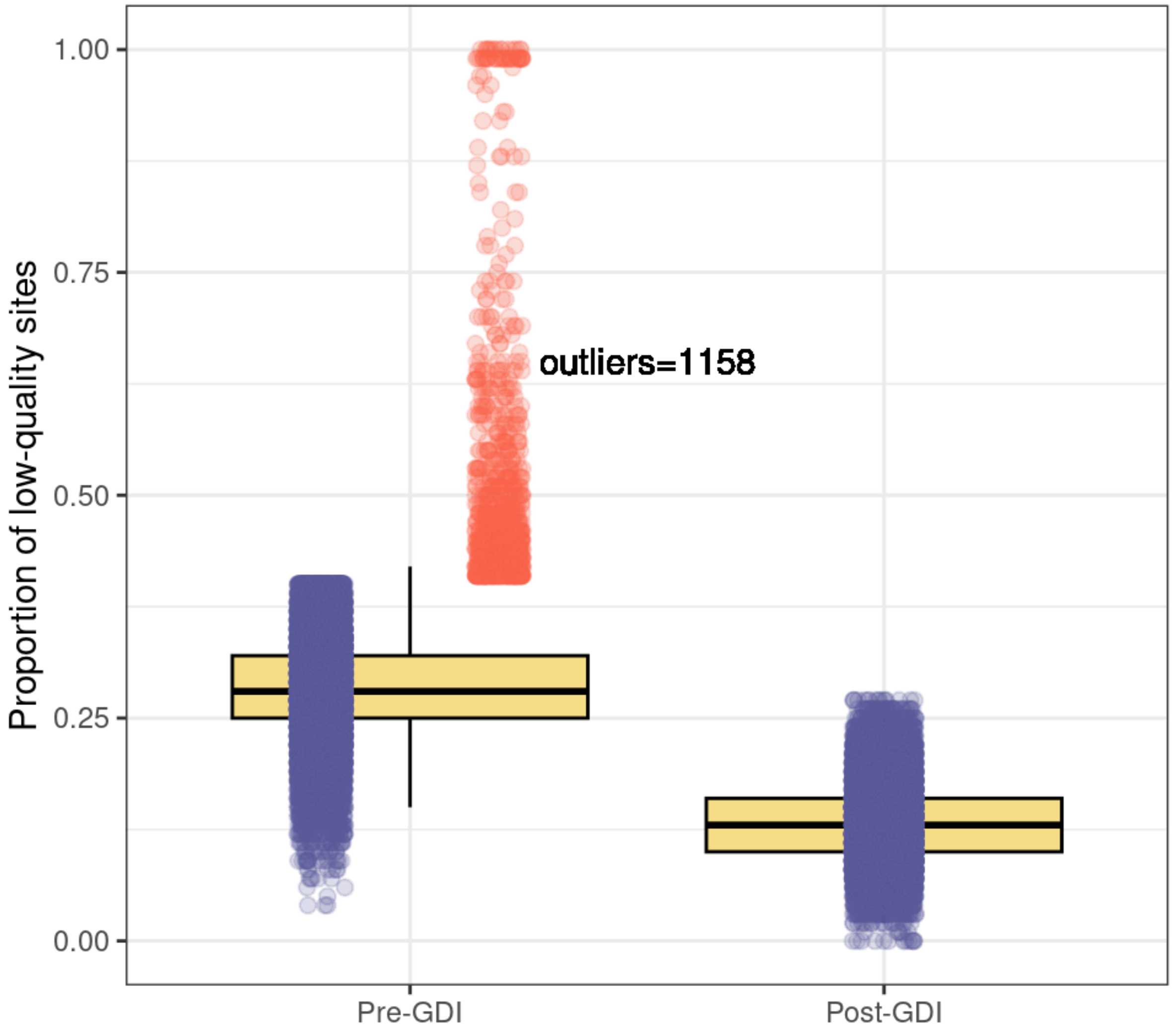

Figure S6 Distribution of LQV (Low-Quality Variant) scores before and after the application of the GDI filter. The median proportion of LQV sites decreases from 0.28 to 0.13 after the removal of low-quality variants in more than 30% of the samples, resulting in an average reduction of 54.1% in LQV scores across the dataset. The standard deviation of 6.2% indicates the variability of individual sample percentage differences from the average reduction.

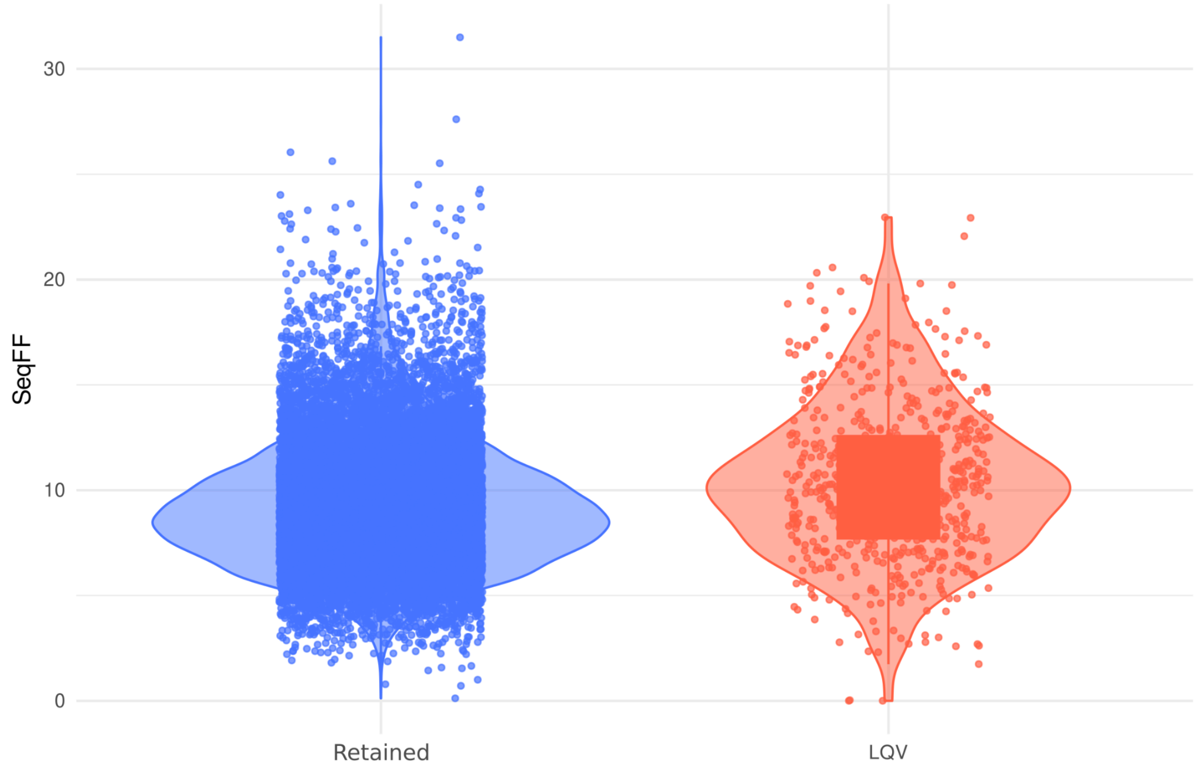

Figure S7 Comparison of fetal fraction (SeqFF) distribution between retained and LQV-removed samples.

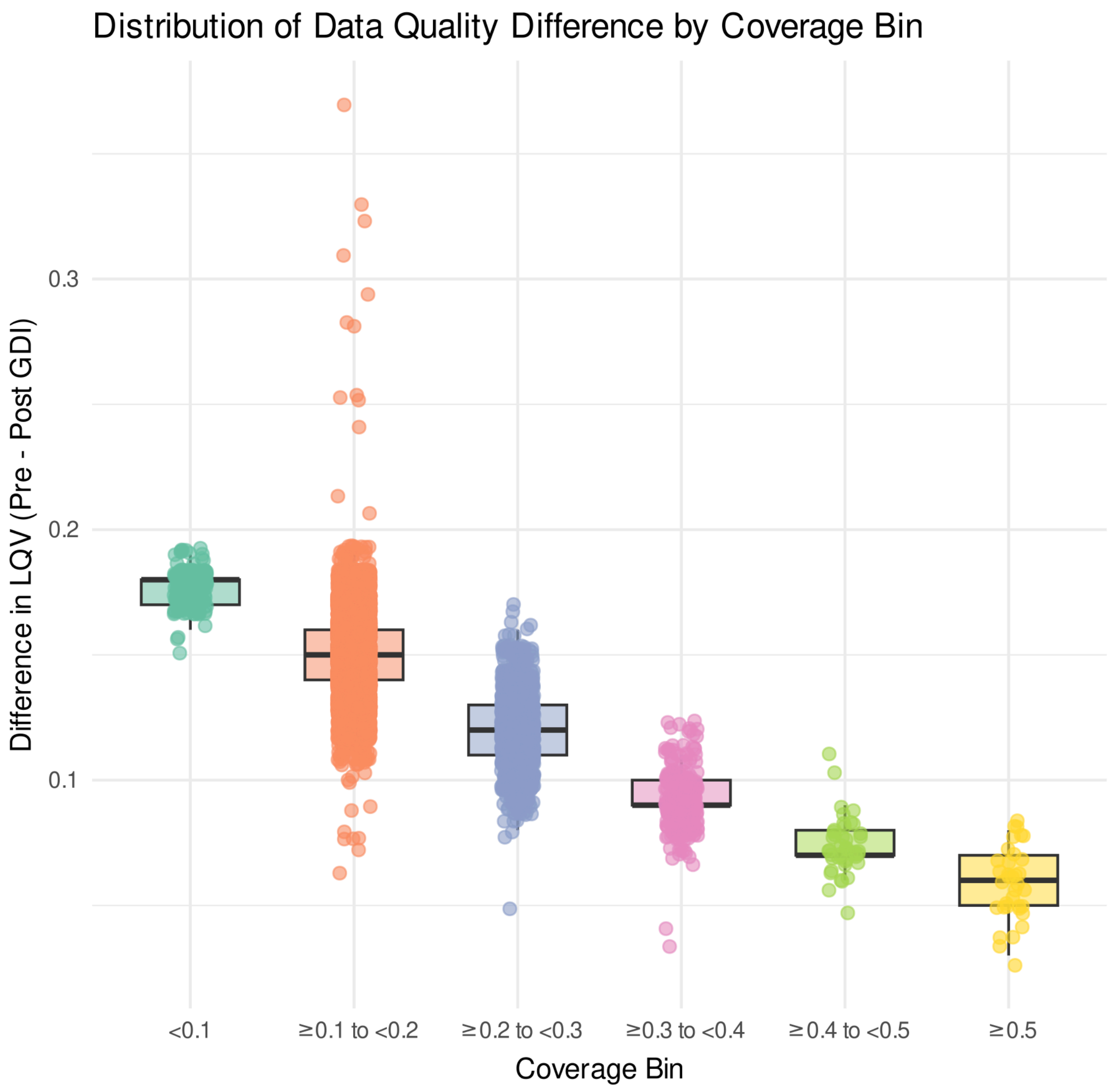

Figure S8 Boxplots displaying the distribution of differences in data quality (LQV pre-GDI - LQV post-GDI) across different coverage bins for a total of 27,354 samples. Each boxplot represents the distribution of individual sample differences within each coverage bin.

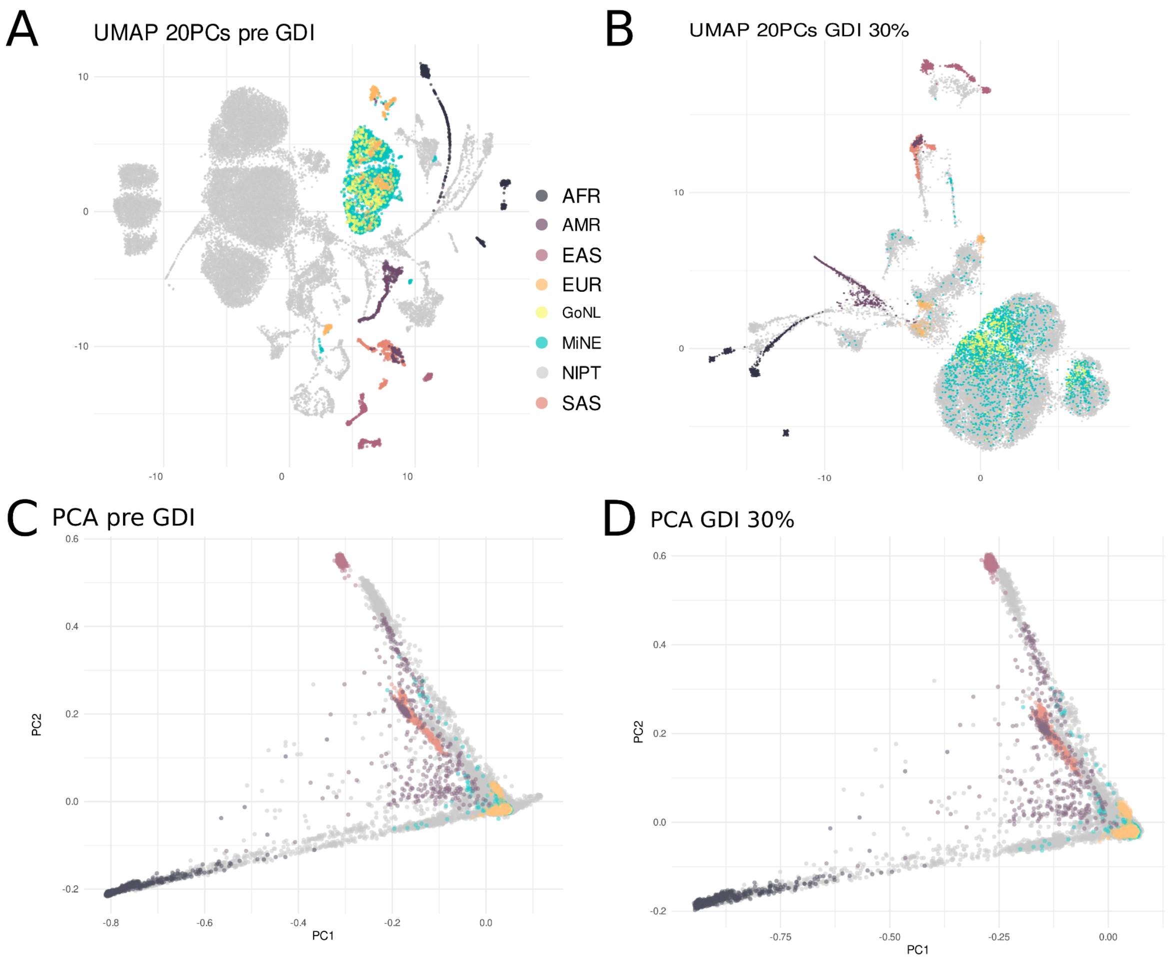

Figure S9 Batch effect removal. All plots NIPS samples, 2.495 samples from the 1000 Genomes Project, 3.643 samples from the MinE dataset, and 498 samples from the GoNL dataset. Panel A shows a UMAP based on 20 principal components (PCs) prior to the application of the GDI filter. Panel B is the result of the cutof used to remove variants with low quality in more than 30% (1.934.218 variants removed) of the NIPS samples. Panel C shows the first two PCs before the application of the GDI filter, while panel D is the resulting filtered dataset. The images are derived from LD-pruned data, retaining overlapping sites across all datasets, with the following counts: 473.352 variants prior to the application of the GDI filter, and 144.935 variants after applying a GDI cutoff of 30%. UMAP was generated using the same input file employed for plotting the UMAP in Figure 4 (panel B), using the same seed; the variation in shape is due to being produced on a different machine.

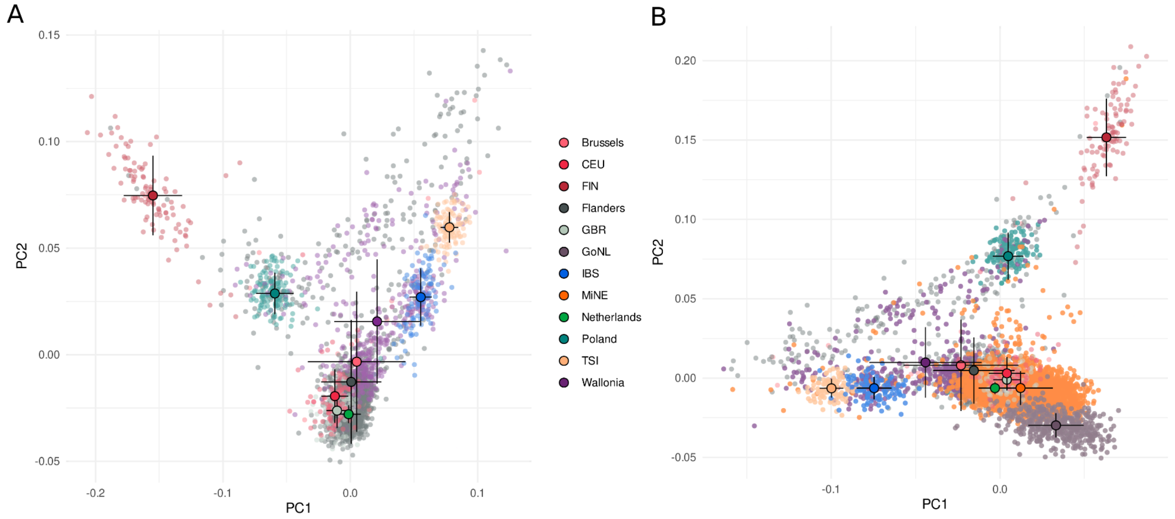

Figure S10 Principal component analysis was conducted using imputed NIPS samples with known labels alongside European samples from the 1000 Genomes Project (**A**), as well as samples from the GoNL and the MinE datasets (**B**). For improved clarity, each plot is also presented as an average PCA, where each group is represented by an average value along with standard deviation bars for both PCs.

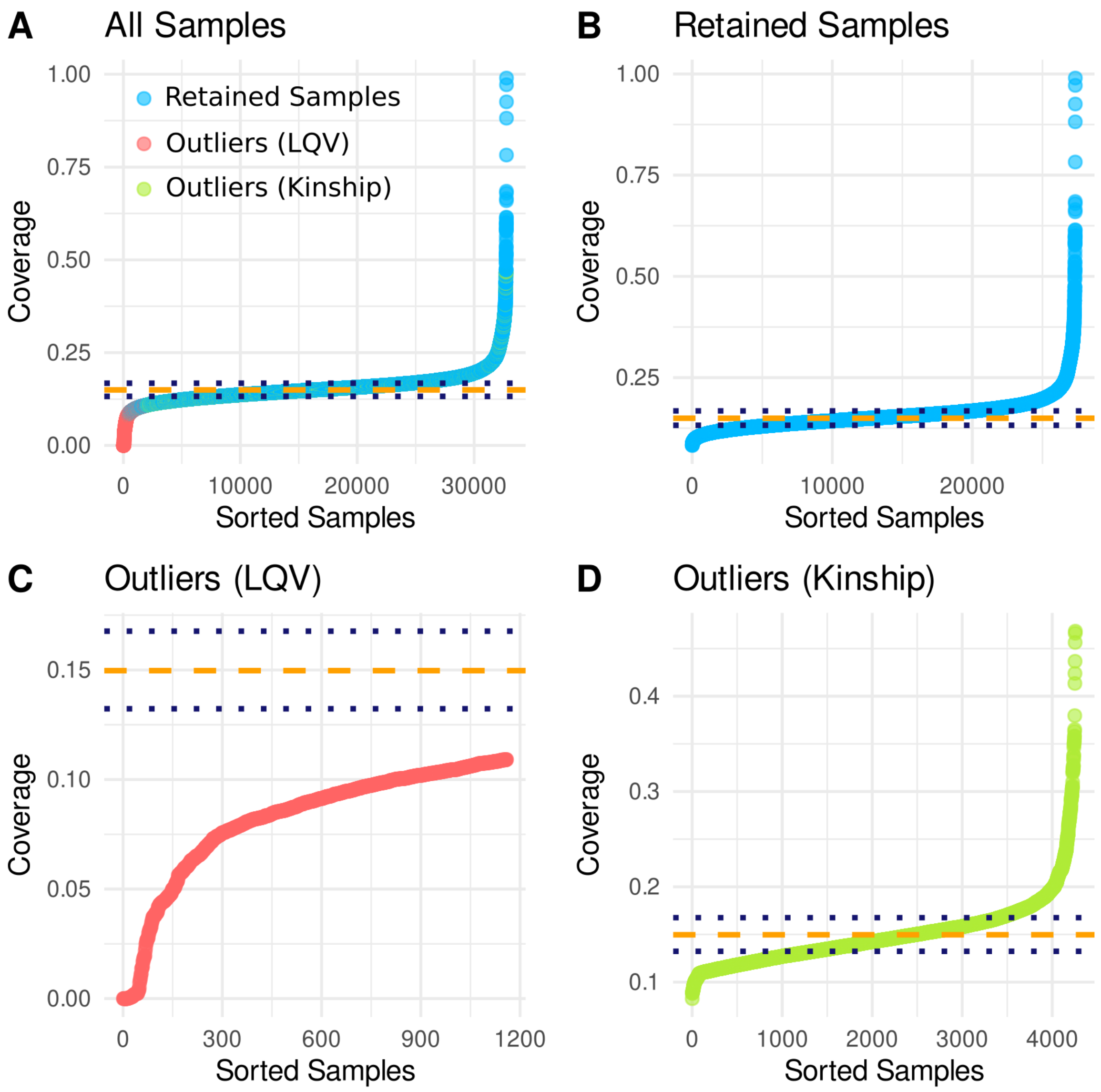

Figure S11 Coverage distribution of 32,769 imputed NIPS samples. Samples are sorted by coverage. The three categories of retained samples, outliers identified through LQV filtering, and outliers based on kinship analysis are displayed together in Panel A and separately in Panels B, C, and D for improved clarity. The median (~0.15x), Q1 (~0.13x), and Q3 (~0.17x) coverage values, calculated for the entire dataset, are indicated in each panel.

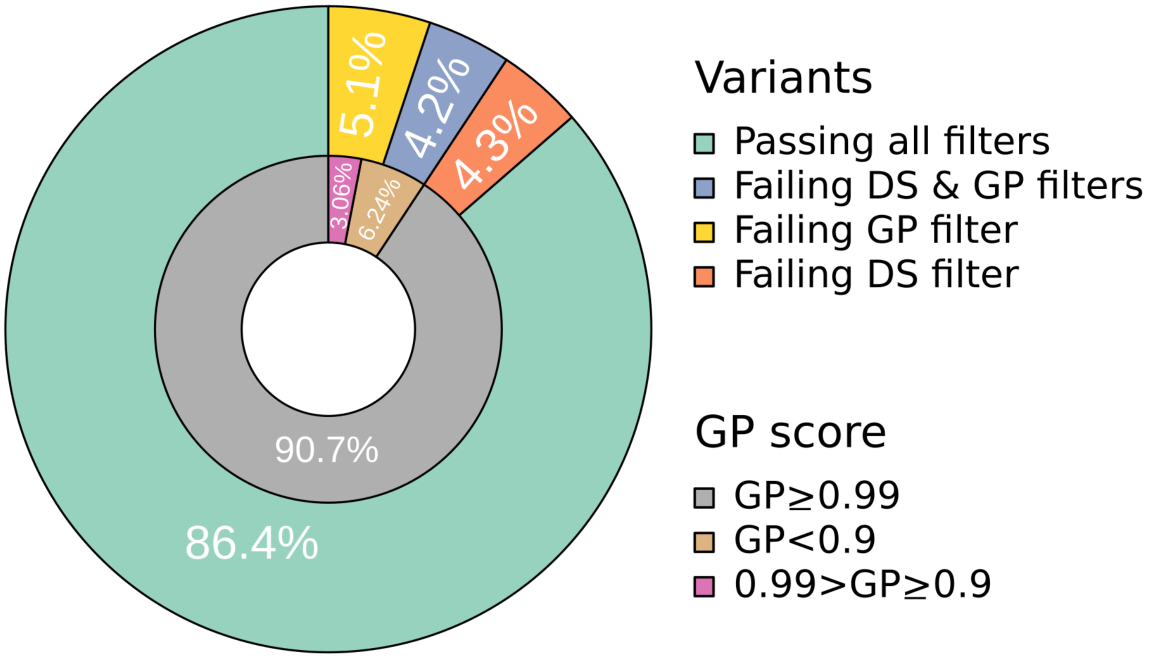

Figure S12 Average distribution of variants after applying the GDI filter on 27.354 NIPS samples. The outer ring shows the percentage of variants in different categories, while the inner ring represents the distribution based on the GP values.
